# Recurrent, Nonrecurrent, and De Novo Membranous Nephropathy After Kidney Transplantation: A Systematic Review and Meta-Analysis

**DOI:** 10.1016/j.xkme.2026.101284

**Published:** 2026-02-06

**Authors:** Thanyarat Phumthian, Veerapat Wattanasatja, Aschariya Wipattanakitcharoen, Thunyatorn Wuttiputhanun, Asada Leelahavanichkul, Natavudh Townamchai, Yingyos Avihingsanon, Suwasin Udomkarnjananun

**Affiliations:** 1 Division of Nephrology, Department of Medicine, Faculty of Medicine, Chulalongkorn University and King Chulalongkorn Memorial Hospital, The Thai Red Cross Society, Bangkok, Thailand; 2 Department of Internal Medicine, Ubon Ratchathani University Hospital and College of Medicine and Public Health, Ubon Ratchathani University, Ubon Ratchathani, Thailand; 3 Department of Internal Medicine, Sunpasitthiprasong Hospital, Ubon Ratchathani, Thailand; 4 Excellence Center for Organ Transplantation (ECOT), King Chulalongkorn Memorial Hospital, The Thai Red Cross Society, Bangkok, Thailand; 5 Center of Excellence on Translational Research in Inflammation and Immunology (CETRII), Department of Microbiology, Faculty of Medicine, Chulalongkorn University, Bangkok, Thailand; 6 Immunology Unit, Department of Microbiology, Chulalongkorn University, Bangkok, Thailand; 7 Renal Immunology and Renal Transplant Center of Excellence, Faculty of Medicine, Chulalongkorn University, Bangkok, Thailand

**Keywords:** Kidney transplantation, membranous nephropathy, meta-analysis, prevalence, recurrent glomerular disease, risk factors, systematic review, treatment

## Abstract

**Rationale & Objective:**

Recurrent and de novo membranous nephropathy (MN) are significant complications after kidney transplantation, yet prevalence, risk determinants, and treatment outcomes have not been comprehensively quantified.

**Study Design:**

Systematic review and meta-analysis.

**Setting & Participants:**

Kidney transplant recipients with native-kidney MN (recurrent or nonrecurrent) and recipients with de novo MN.

**Selection Criteria for Studies:**

PubMed, Scopus, and the Cochrane Library were searched through June 30, 2025; studies comparing risk factors and outcomes among these groups were eligible for meta-analyses.

**Data Extraction:**

Study characteristics, demographics, transplant features, outcomes including remission and allograft loss.

**Analytic Approach:**

Random-effects meta-analyses calculated weighted mean differences or pooled ORs for comparisons between recurrent versus nonrecurrent or de novo MN, allograft outcomes, and response to rituximab.

**Results:**

The included studies comprised a total of 2,259 kidney transplant recipients with recurrent (28%), nonrecurrent (61%), or de novo MN (11%). Recurrence prevalence was 39% (95% CI, 28%-50%) in studies with protocol biopsies versus 25% (95% CI, 20%-29%) without protocol biopsies (*P* = 0.046). Compared with nonrecurrence, recurrent MN was linked to older recipient age, shorter dialysis vintage and interval from native MN to dialysis, living-donor grafts, and higher pretransplant antiphospholipase A2 receptor antibody titer. Mycophenolic acid and prednisolone use was protective against recurrent MN. De novo MN carried a higher associated rejection than recurrent MN (OR, 2.30; 95% CI, 1.16-4.58). Rituximab increased remission odds (OR, 4.90; 95% CI, 1.70-14.13). Meta-regression demonstrated a significant decline in allograft loss rates over time following MN recurrence.

**Limitations:**

Substantial heterogeneity and small-study effects in some variables; magnitudes should be interpreted cautiously.

**Conclusions:**

MN recurs in approximately one-third of recipients. Protocol biopsy should be utilized in recipients with history of native MN. Rituximab emerges as the preferred first-line treatment for recurrent MN.

## Plain-language Summary

Recurrent glomerular disease after kidney transplantation is a challenging posttransplant complication, and recurrent membranous nephropathy (MN) is among the most common entities. Prior evidence has established pretransplant antiphospholipase A2 receptor antibodies as a risk factor for recurrence; however, other risk factors and treatment outcomes have not been comprehensively assessed. This study is the first to include all available evidence on posttransplant MN and shows that protocol biopsy increases detection of recurrent MN and is associated with a lower risk of allograft loss. In addition to identifying several risk factors for recurrence, the analysis demonstrates that rituximab therapy yields a significantly higher remission rate than non-rituximab treatment in recurrent MN.

## Introduction

Advances in kidney transplantation have led to substantial reductions in acute allograft rejection and improvements in long-term graft survival.^1^^,^^2^ Nevertheless, recurrence of glomerular disease remains a major contributor to graft loss.^3^ Among primary glomerulopathies, membranous nephropathy (MN) is particularly notorious for its propensity to recur posttransplant, alongside other entities such as IgA nephropathy, primary focal segmental glomerulosclerosis, and membranoproliferative glomerulonephritis, notably complement-mediated forms (eg, C3 glomerulonephritis) and monoclonal immunoglobulin-mediated diseases.^4^

The impact of MN on allograft survival has been inconsistently reported. Some studies have demonstrated significantly higher rates of graft loss in recipients with recurrent MN compared with those without recurrence,^5^^,^^6^ whereas others have found no difference in long-term outcomes.^7^^,^^8^ MN in kidney transplant recipients (KTRs) can be stratified into 3 distinct populations: recurrent MN in recipients whose kidney failure was due to native MN and who experience histologic recurrence, nonrecurrent MN recipients with native MN who do not develop recurrence, and de novo MN recipients whose kidney failure was caused by other etiologies but who develop MN after transplantation. Although numerous cohort studies, case series, and case reports have explored these groups, examining epidemiology, risk factors, immunologic biomarkers (eg, antiphospholipase A2 receptor [PLA2R] antibodies), treatment responses, and outcomes, the reported prevalence of recurrence and associated effect sizes remain heterogeneous and have not been synthesized quantitatively.

To address these gaps, we conducted a systematic review and meta-analysis of MN after kidney transplantation, encompassing all 3 patient categories. Our objectives were to estimate the pooled prevalence of recurrent MN and identify clinical and immunologic risk factors for recurrence. The efficacy of key therapies, with a focus on anti-CD20 antibody (rituximab), and patient and graft outcomes were assessed. By synthesizing data from case reports, case series, and cohort studies, this work aims to provide clinicians with a unified, quantitative synthesis of MN epidemiology and management in the kidney transplantation setting, ultimately informing risk stratification, monitoring protocols, and individualized treatment approaches.

## Methods

### Data Source and Search

This systematic review was conducted in accordance with the Preferred Reporting Items for Systematic Reviews and Meta-Analyses (PRISMA) guidelines.^9^ We searched PubMed, Scopus, and the Cochrane Library through June 30, 2025. In PubMed, the terms “Kidney Transplantation” AND (“Glomerulonephritis, Membranous” OR “Nephrotic Syndrome”) were used, while Scopus searches employed TITLE-ABS-KEY (kidney AND transplantation) AND TITLE-ABS-KEY (membranous AND nephropathy). In the Cochrane Library, MeSH descriptors for [Membranous Nephropathy] and [Kidney Transplantation] were combined with equivalent free-text terms. The review protocol was registered in PROSPERO (CRD420251108867). Finally, the reference lists of all relevant articles were screened to identify additional eligible studies.

### Study Selection

This systematic review and meta-analysis sought to consolidate all available evidence on MN after kidney transplantation, specifically recurrent, nonrecurrent, and de novo forms, by including every eligible study design, from randomized trials (if available) and observational cohorts to case-control studies, case series, and case reports. Case series report multiple cases within a single study and may include ≥1 of the groups of interest. In contrast, case reports describe a single patient in 1 of the 3 designated groups. Eligible publications had to present at least one pertinent clinical dimension of posttransplant MN, such as baseline transplant characteristics, alloimmune (eg, human leukocyte antigen [HLA] mismatches) or autoimmune immunologic assessments (eg, anti-PLA2R antibody status), laboratory data at diagnosis, details of induction or maintenance immunosuppression, therapeutic interventions administered, or subsequent graft and patient outcomes. Only English-language studies involving adult recipients (aged ≥18 years) were considered, while animal experiments and in vitro investigations were excluded. Two reviewers (T.P. and V.W.) independently screened titles and abstracts, evaluated full texts for eligibility, and resolved any discrepancies through consensus with the full author group.

### Data Extraction and Quality Assessment

Data on publication characteristics, including author names, year of publication, country of origin, and journal, were extracted. Transplant recipient information comprised age, sex, dialysis vintage, interval from native MN to kidney failure, HLA mismatches, induction regimens, and maintenance immunosuppressive therapies. Variables specific to MN included the time from transplantation to MN diagnosis (stratified by recurrent versus de novo disease), serum creatinine and proteinuria at diagnosis, biopsy indication (protocol versus for-cause), anti-PLA2R antibody titers and positivity rates, histopathologic findings, treatments administered, and graft outcomes.

Quality assessment of cohort and case-control studies was performed using the Newcastle-Ottawa Scale, which evaluates selection (up to 4 stars), comparability (up to 2 stars), and outcome assessment (up to 3 stars) for a maximum score of 9, with higher scores indicating lower risk of bias.^10^ Case reports, case series, and cross-sectional studies were appraised with the Joanna Briggs Institute Critical Appraisal Checklists; each item was rated as “Yes,” “No,” “Unclear,” or “Not Applicable,” and a greater number of affirmative responses reflected higher methodological quality.^11^

### Data Synthesis and Analysis

Data from each study were pooled within the recurrent MN, nonrecurrent MN, and de novo MN groups and summarized as mean ± standard deviation or as percentages of transplant recipients. For the meta-analyses, we used random-effects models with inverse-variance weighting; between-study variance (τ^2^) was estimated using the Paule–Mandel method, and 95% confidence intervals (CIs) were calculated with Hartung–Knapp–Sidik–Jonkman adjustments. We performed available-case analyses. Studies that did not report sufficient data to compute an effect size (eg, missing dispersion for continuous outcomes, incomplete 2 × 2 data) were excluded from the corresponding meta-analyses and summarized narratively. Dichotomous outcomes were pooled as log odds ratios (ORs); when a study arm contained no events, we applied a 0.5 continuity correction. Continuous outcomes were summarized as weighted mean differences (WMDs), and standardized mean differences were applied to anti-PLA2R antibody titers to accommodate variability in assay units and methods across centers. We included only cohort studies and case series that reported the same variables for at least 2 of the 3 MN groups, using these data to compare factors associated with recurrent versus nonrecurrent or de novo MN and to assess the risk of kidney allograft loss. Because immunosuppressive regimens and treatment paradigms for posttransplant MN have evolved over time, we performed meta-regression to examine the relationship between graft survival and year of publication.

For prevalence outcomes, proportions were pooled under a random-effects model after application of the Freeman–Tukey double-arcsine transformation, with pooled estimates back-transformed to the proportion scale; a prespecified subgroup analysis was conducted contrasting studies with protocol biopsies versus those without. Only cohort studies and case series that reported the total number of native MN cases and the number of recurrences were included in the meta-analysis of prevalence.

Heterogeneity was assessed using Cochran’s Q test (*P* < 0.10 indicating significant heterogeneity) and quantified by the *I*^2^ statistic (values >50% denoting substantial heterogeneity).^12^ Potential publication bias was evaluated by visual inspection of funnel plots and by Egger’s regression asymmetry test (*P* < 0.05 suggesting significant small-study effects). All statistical analyses were performed in Stata version 19 (StataCorp LLC).

## Results

### Overview of Study Search and Characteristics of MN Posttransplantation

**Figure 1:**
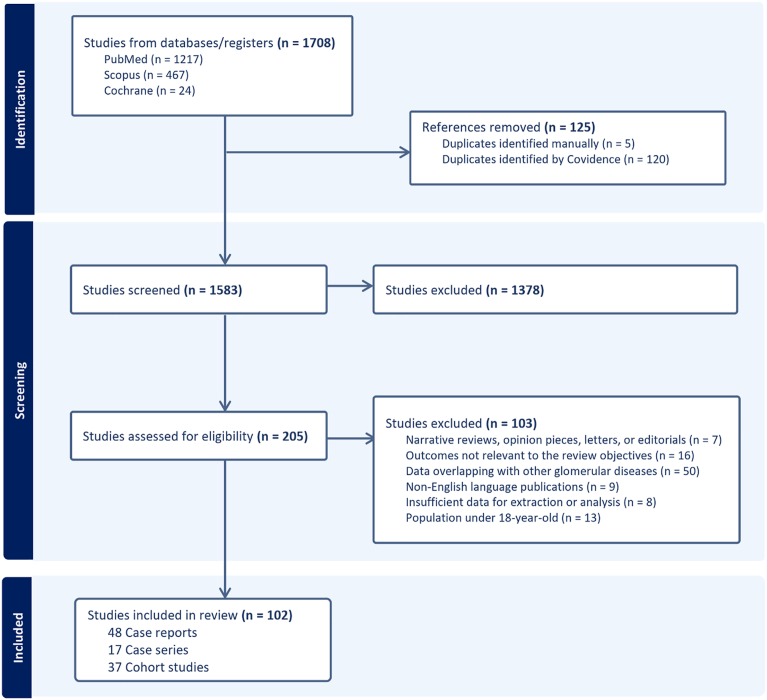
Study search and selection according to PRISMA flowchart.

The predefined search strategy identified 1,708 records from electronic databases. After duplicates were removed and relevance screening was completed, 205 full-text articles underwent review, of which 102 met the inclusion criteria and were included in the analyses (Fig 1). These comprised 37 cohort studies,^6^, ^7^, ^8^^,^^13^, ^14^, ^15^, ^16^, ^17^, ^18^, ^19^, ^20^, ^21^, ^22^, ^23^, ^24^, ^25^, ^26^, ^27^, ^28^, ^29^, ^30^, ^31^, ^32^, ^33^, ^34^, ^35^, ^36^, ^37^, ^38^, ^39^, ^40^, ^41^, ^42^, ^43^, ^44^, ^45^, ^46^ 17 case series,^5^^,^^47^, ^48^, ^49^, ^50^, ^51^, ^52^, ^53^, ^54^, ^55^, ^56^, ^57^, ^58^, ^59^, ^60^, ^61^, ^62^ and 48 case reports.^63^, ^64^, ^65^, ^66^, ^67^, ^68^, ^69^, ^70^, ^71^, ^72^, ^73^, ^74^, ^75^, ^76^, ^77^, ^78^, ^79^, ^80^, ^81^, ^82^, ^83^, ^84^, ^85^, ^86^, ^87^, ^88^, ^89^, ^90^, ^91^, ^92^, ^93^, ^94^, ^95^, ^96^, ^97^, ^98^, ^99^, ^100^, ^101^, ^102^, ^103^, ^104^, ^105^, ^106^, ^107^, ^108^, ^109^, ^110^ Among these, 60 studies examined recurrent MN in recipients whose kidney failure was attributed to native MN,^5^, ^6^, ^7^, ^8^^,^^13^^,^^16^^,^^18^^,^^19^^,^^21^^,^^23^, ^24^, ^25^^,^^27^, ^28^, ^29^^,^^32^^,^^33^^,^^35^, ^36^, ^37^^,^^39^, ^40^, ^41^, ^42^, ^43^^,^^45^^,^^53^^,^^55^, ^56^, ^57^, ^58^, ^59^^,^^63^, ^64^, ^65^^,^^68^^,^^70^, ^71^, ^72^^,^^75^^,^^79^, ^80^, ^81^^,^^83^^,^^85^^,^^86^^,^^88^, ^89^, ^90^, ^91^^,^^95^^,^^98^, ^99^, ^100^, ^101^, ^102^^,^^105^^,^^106^^,^^109^^,^^110^ 29 studies reported on de novo MN,^15^^,^^31^^,^^38^^,^^47^, ^48^, ^49^^,^^52^^,^^60^^,^^62^^,^^66^^,^^67^^,^^69^^,^^73^^,^^74^^,^^76^, ^77^, ^78^^,^^82^^,^^84^^,^^87^^,^^92^, ^93^, ^94^^,^^96^^,^^97^^,^^103^^,^^104^^,^^107^^,^^108^ and 13 studies addressed both recurrent and de novo MN.^14^^,^^17^^,^^20^^,^^22^^,^^26^^,^^30^^,^^34^^,^^44^^,^^46^^,^^50^^,^^51^^,^^54^^,^^61^ Table 1 summarizes the characteristics of the included cohort studies, and Supplementary Table S1 presents those of the case series and case reports; collectively, these sources enrolled 2,259 KTRs (28% recurrent, 61% nonrecurrent, and 11% de novo MN). Two studies^6^^,^^43^ included 2 subcohorts, each of which was analyzed separately. The majority of studies originated from the USA, and 19 studies employed protocol surveillance biopsies to diagnose posttransplant MN.^7^^,^^8^^,^^23^^,^^25^, ^26^, ^27^^,^^29^^,^^30^^,^^32^^,^^35^^,^^39^^,^^43^^,^^57^, ^58^, ^59^^,^^61^^,^^62^^,^^102^^,^^107^ Quality assessment results (Tables S2 and S3) indicated fair to good methodological quality in most of the included studies.

**Table 1:** Characteristics of Cohort Studies Included in the Systematic Review and Meta-analysis

Reference	Year of Publication	Journal	Country	Recurrent MN	De novo MN	Protocol Biopsy	Total Patients Included	Treatment With Rituximab
Morzycka et al^13^	1982	Am J Med	USA	Yes	No	No	7	No
Honkanen et al^14^	1984	Clin Nephrol	Finland	Yes	Yes	No	7	No
Ward et al^15^	1988	Transplantation	USA	No	Yes	No	14	No
O’Meara et al^16^	1989	Nephrol Dial Transplant	Ireland	Yes	No	No	10	No
Schwarz et al^17^	1991	Am J Kidney Dis	Germany	Yes	Yes	No	15	No
Couchoud et al^18^	1995	Transplantation	France	Yes	No	No	19	No
Odorico et al^19^	1996	Transplantation	USA	Yes	No	No	64	No
Morales et al^20^	1997	Curr Opin Nephrol Hypertens	Spain	Yes	Yes	No	15	No
Hariharan et al^21^	1998	Am J Kidney Dis	USA	Yes	No	No	11	No
Hariharan et al^22^	1999	Transplantation	USA	Yes	Yes	No	16	No
Briganti et al^23^	2002	N Engl J Med	ANZDATA	Yes	No	Yes	81	No
Ibrahim et al^24^	2006	Transplantation	USA	Yes	No	No	19	No
Dabade et al^25^	2008	Am J Transplant	USA	Yes	No	Yes	19	No
Aline-Fardin et al^26^	2009	Transplant Proc	France	Yes	Yes	Yes	15	No
El-Zoghby et al^27^	2009	Am J Transplant	USA	Yes	No	Yes	8	Yes
Moroni et al^28^	2010	Nephrol Dial Transplant	Italy	Yes	No	No	35	Yes
Sprangers et al^29^	2010	Clin J Am Soc Nephrol	USA	Yes	No	Yes	34	Yes
Debiec et al^30^	2011	Am J Transplant	France	Yes	Yes	Yes	25	No
Honda et al^31^	2011	Clin Transplantation	Japan	No	Yes	No	17	No
Rodriguez et al^32^	2012	Am J Transplant	USA	Yes	No	Yes	29	Yes
Kennedy et al^33^	2013	Int J Nephrol	Ireland	Yes	No	No	32	Yes
Larsen et al^34^	2013	Transplantation	USA	Yes	Yes	No	22	No
Kattah et al^35^	2015	Am J Transplant	USA	Yes	No	Yes	26	Yes
Quintana et al^36^	2015	Transplantation	Spain	Yes	No	No	21	Yes
Spinner et al^37^	2015	Am J Nephrol	USA	Yes	No	No	48	Yes
Wen et al^38^	2016	Medicine	China	No	Yes	No	23	No
Grupper et al^39^	2016	Transplantation	USA	Yes	No	Yes	63	Yes
Gupta et al^40^	2016	Clin Transplant	USA	Yes	No	No	16	Yes
Jiang et al^41^	2018	BMC Nephrol	ANZDATA	Yes	No	No	309	No
Singh et al^42^	2019	Clin Transplant	USA	Yes	No	No	81	No
Berchtold et al^43^	2021	Kidney Int	Europe, Canada, USA, UK	Yes	No	Yes	145	No
Chung et al^7^	2022	Transplant Direct	ANZDATA	Yes	No	Yes	199	No
Buxeda et al^44^	2023	Clin Kidney J	Spain	Yes	Yes	No	75	Yes
Chukwu et al^45^	2023	Clin Transplant	UK	Yes	No	No	16	No
Cremoni et al^6^	2024	Kidney Int Rep	France	Yes	No	No	137	No
Hullekes et al^8^	2024	Am J Transplant	Multicontinental	Yes	No	Yes	194	Yes
Khorsandi et al^46^	2024	Front Nephrol	USA	Yes	Yes	No	26	No

Abbreviation: MN, membranous nephropathy.

Table 2 presents the characteristics of KTRs with recurrent MN, nonrecurrent MN, and de novo MN. Pooled estimates were derived from cohort studies, case series, and case reports to provide an overview of patient features in each group. Mean follow-up durations were 76.8 ± 54.2 months for the recurrent MN group, 89.4 ± 49.3 months for the nonrecurrent MN group, and 57.6 ± 48.2 months for the de novo MN group. Because some studies employed single-arm designs (reporting data for only 1 of the 3 groups), formal statistical comparisons were not conducted. Notable differences nevertheless emerged: the proportion of living-donor transplants was higher in the recurrent MN group than in the nonrecurrent MN group (51.2% vs 30.8%), recipients without recurrence exhibited a longer interval from native MN diagnosis to end-stage kidney disease (116.1 ± 51.0 months vs 76.3 ± 49.6 months), and pretransplant anti-PLA2R titers (148 ± 402 relative units [RU]/mL vs 426 ± 1,464 RU/mL) and anti-PLA2R positivity rates (23.7% vs 71.2%) were lower in the nonrecurrent MN group than in the recurrent MN group.

**Table 2:** Summary of Baseline Characteristics of KTR With Recurrent Native MN, No Recurrence of Native MN, and De Novo MN

Variables	Recurrent MN		No Recurrent MN		De Novo MN	
	Values^a^	Studies Included (Total KTRs Included)	Values^a^	Studies Included (Total KTRs Included)	Values^a^	Studies Included (Total KTRs Included)
Age at transplantation, y	50.9 ± 11.7	59 (471)	50.1 ± 12.7	20 (749)	39.3 ± 13.9	39 (211)
Male sex	78.3%	58 (456)	73.1%	19 (735)	62.1%	37 (202)
Living donor KT	51.2%	34 (385)	30.8%	18 (735)	73.4%	10 (64)
Dialysis before KT	84.6%	30 (195)	81.0%	10 (385)	97.4%	16 (37)
Dialysis vintage, mo	23.9 ± 22.3	31 (257)	26.7 ± 27.3	14 (672)	38.5 ± 35.4	14 (35)
Time from native MN diagnosis to kidney failure, mo	76.3 ± 49.6	23 (156)	116.1 ± 51.0	9 (379)	N/A	N/A
HLA mismatch (sum of A, B, DR)	3.2 ± 0.9	6 (117)	3.4 ± 1.0	3 (213)	2.5 ± 1.5	4 (63)
Pre-KT anti-PLA2R titer, RU/mL	426 ± 1464	8 (58)	148 ± 402	6 (37)	N/A	N/A
Patients with positive pre-KT anti-PLA2R	71.2%	16 (132)	23.7%	9 (194)	N/A	N/A
T-cell depleting induction	57.8%	29 (344)	35.7%	11 (530)	70.6%	5 (50)
Tacrolimus use	71.6%	24 (232)	70.9%	11 (522)	100%	9 (9)
MPA use	78.2%	28 (238)	83.0%	10 (511)	100%	6 (6)
Prednisolone use	82.2%	43 (315)	89.7%	9 (457)	90.9%	25 (98)
Time from KT to MN, mo	29.2 ± 38.0	66 (534)	N/A	N/A	48.8 ± 48.5	37 (204)
Serum Cr at post-KT MN diagnosis, mg/dL	2.0 ± 1.5	28 (176)	N/A	N/A	2.1 ± 1.4	25 (106)
Urine protein at post-KT MN diagnosis, mg/d	4272 ± 7162	36 (189)	N/A	N/A	4990 ± 4937	24 (94)
Graft loss	30.6%	30 (278)	16.4%	12 (586)	56.2%	16 (146)
Follow-up time, mo	76.8 ± 54.2	27 (306)	89.4 ± 49.3	10 (256)	57.6 ± 48.2	12 (57)

Abbreviations: Cr, creatinine; HLA, human leukocyte antigen; KT, kidney transplantation; KTR, kidney transplantation recipient; MN, membranous nephropathy; MPA, mycophenolic acid; N/A, not available; PLA2R, phospholipase A2 receptor; RU, relative units.

a Mean ± standard deviation for continuous variables, percentage for categorical variables.

### Meta-analysis of Recurrent MN Prevalence

**Figure 2:**
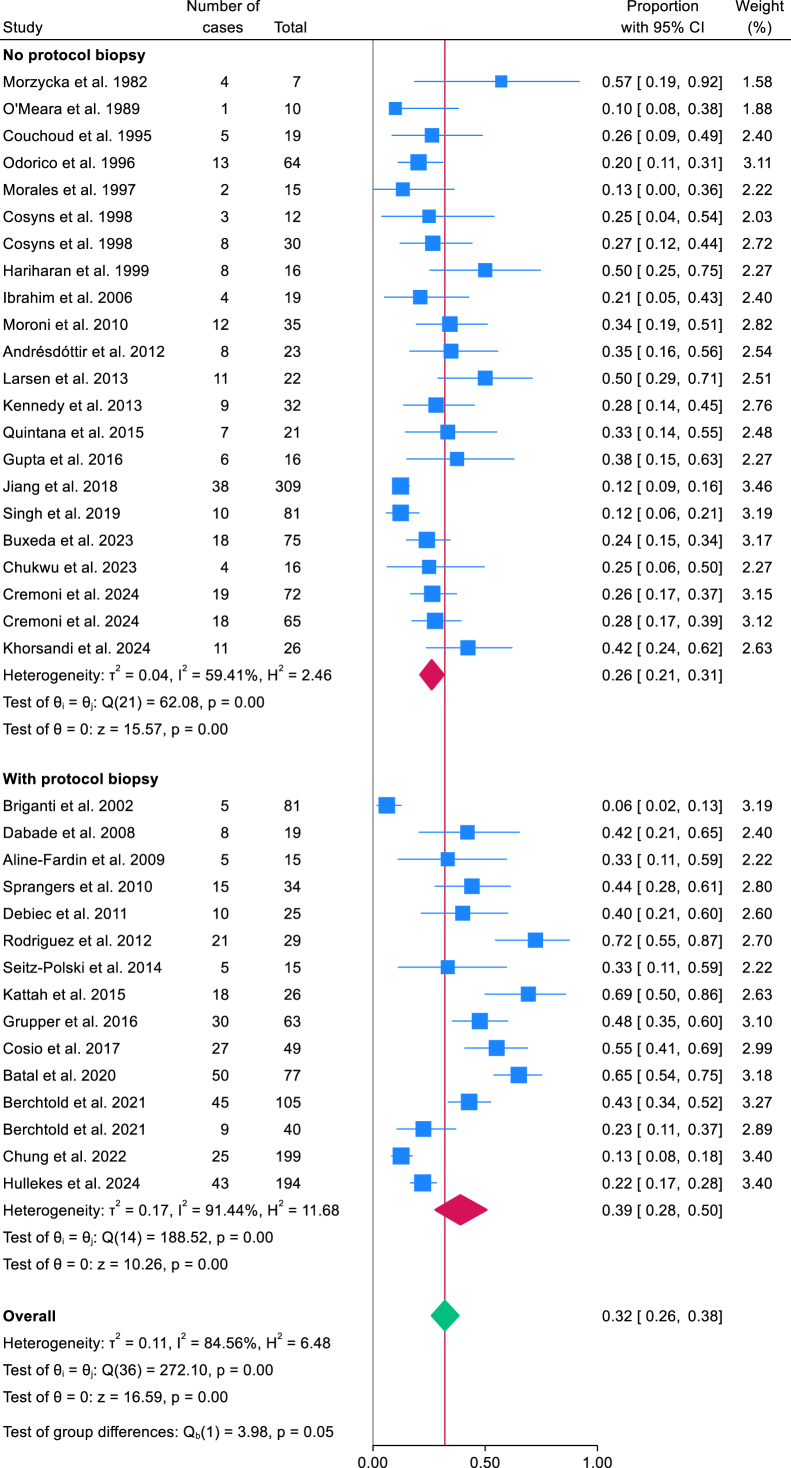
Forest plots of recurrent membranous nephropathy prevalence with subgroup analysis stratified by biopsy indication: protocol biopsies versus no protocol biopsies. CI, confidence interval.

Figure 2 presents the meta-analysis of recurrent MN prevalence among KTRs whose native kidney failure was attributed to MN. Thirty-seven studies^5^, ^6^, ^7^, ^8^^,^^13^^,^^16^^,^^18^, ^19^, ^20^^,^^22^, ^23^, ^24^, ^25^, ^26^^,^^28^, ^29^, ^30^^,^^32^, ^33^, ^34^, ^35^, ^36^^,^^39^, ^40^, ^41^, ^42^, ^43^, ^44^, ^45^, ^46^^,^^55^^,^^57^^,^^59^^,^^61^ reported 535 recurrences among 1,956 such recipients. The overall pooled prevalence was 32% (95% CI, 26%-38%; *P* < 0.001), with substantial heterogeneity (*I*^2^ = 85%; Q-test *P* < 0.001). Subgroup analysis showed that studies employing protocol biopsies exhibited a higher pooled prevalence of recurrent MN (39%; 95% CI, 28%-50%) compared with studies without protocol biopsies (26%; 95% CI, 21%-31%), with a statistically significant between-group difference (*P* = 0.046). The corresponding funnel plot (Fig S1) demonstrated no significant asymmetry (Egger’s test *P* = 0.11).

### Meta-analysis of Factors Associated With Recurrent MN Compared With Nonrecurrent MN

**Figure 3:**
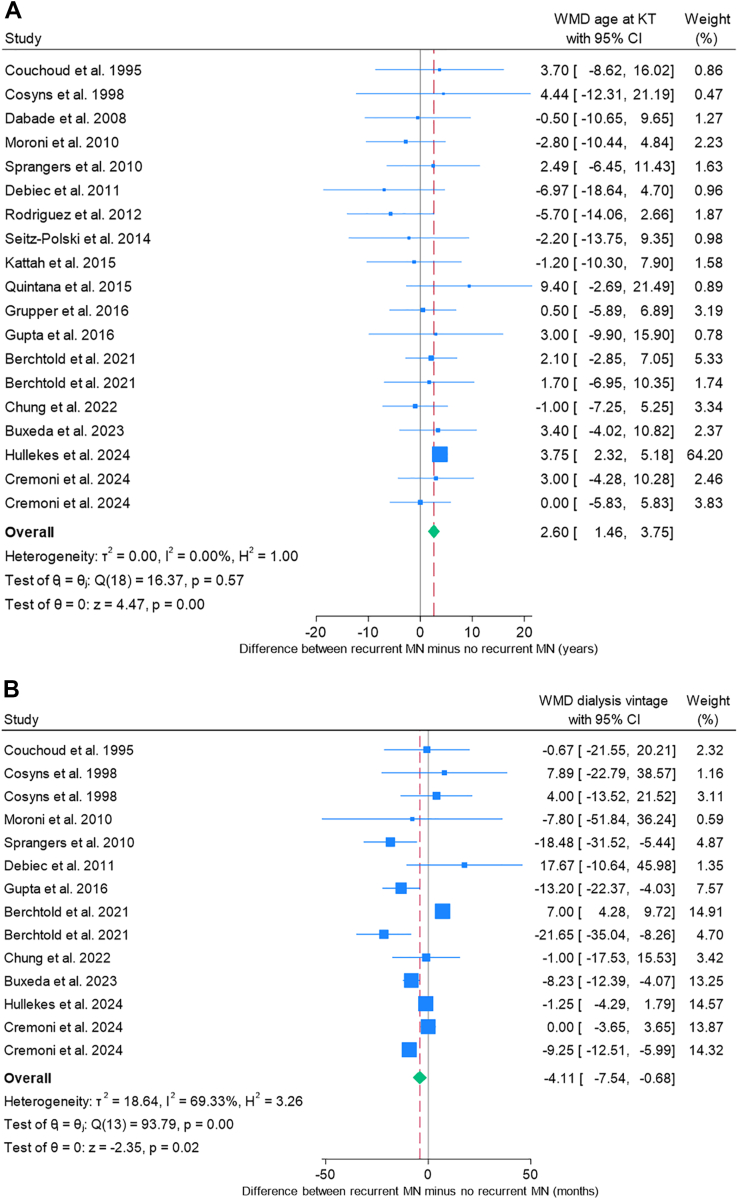

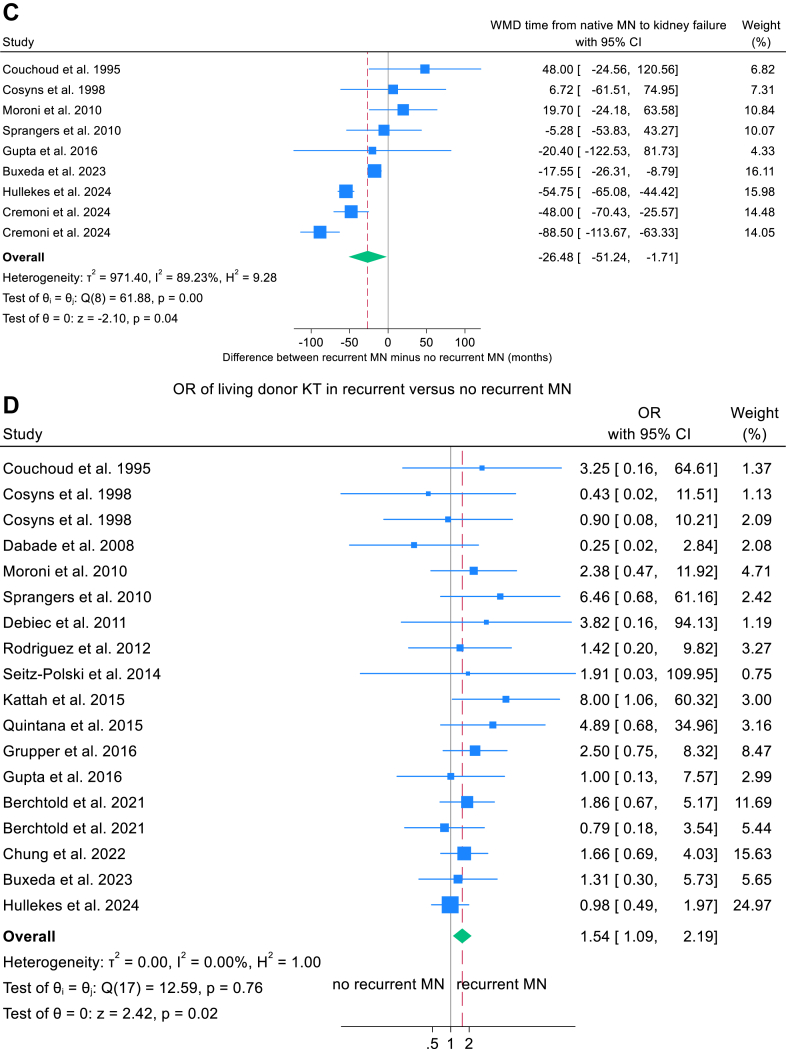
Forest plots of baseline characteristics in KTR associated with recurrent MN. (A) WMD in age at transplantation. (B) WMD in dialysis vintage. (C) WMD in interval from native MN to kidney failure. (D) Pooled OR for living donor kidney transplantation. CI, confidence interval; KTR; kidney transplant recipient, MN, membranous nephropathy; OR; odds ratio, WMD; weighted mean difference.

Meta-analyses were restricted to studies reporting sufficient data on both recurrent and nonrecurrent MN groups and are summarized in Table 3. Baseline demographic factors associated with an increased risk of recurrent MN included older age at transplantation (WMD, 2.6 years; 95% CI, 1.5-3.8; *P* < 0.001), shorter dialysis vintage (WMD, −4.1 months; 95% CI, −7.5 to −0.7; *P* = 0.019), shorter interval from native MN diagnosis to kidney failure (WMD, −26.5 months; 95% CI, −51.3 to −1.7; *P* = 0.036), and receipt of a living-donor transplant (OR, 1.54; 95% CI, 1.09-2.19; *P* = 0.015) (Fig 3A-D). Corresponding funnel plots are presented in Fig S2A-D, and forest and funnel plots for nonsignificant variables are shown in Fig S3A-H.

**Table 3:** Meta-analysis of Factors Associated With Recurrent MN Versus Nonrecurrence in KTRs Whose Kidney Failure Was Caused by Native MN

Variables	Weighted Mean Difference (WMD)	95% CI	*P*	Studies Included (Total KTRs Included)	*I*^2^ Index (%)	Q-Test *P*	Egger’s Test *P*
Age at KT, y	2.6	1.5-3.8	<0.001	19 (1,051)	0%	0.57	0.02
Dialysis vintage, mo	−4.1	−7.5 to −0.7	0.02	14 (908)	69%	<0.001	0.97
Time from native MN to kidney failure, mo	−26.5	−51.3 to −1.7	0.04	9 (518)	89%	<0.001	0.06
Pre-KT anti-PLA2R titer^a^	1.65	0.95-2.34	<0.001	6 (243)	77%	<0.001	0.008
Variables	Pooled Odds Ratio (OR)	95% CI	*P*	Studies Included (Total KTRs Included)	*I*^2^ Index (%)	Q-Test *P*	Egger’s Test *P*
Male sex	1.32	0.90-1.91	0.15	18 (1,032)	7%	0.20	0.39
Living donor KT	1.54	1.09-2.19	0.02	18 (944)	0%	0.76	0.45
Patients with positive pre-KT anti-PLA2R	9.79	3.88-24.66	<0.001	8 (298)	50%	0.05	0.30
T-cell depleting induction therapy	1.02	0.68-1.52	0.93	11 (733)	0%	0.60	0.40
Tacrolimus use	0.70	0.36-1.38	0.31	10 (714)	38%	0.16	0.97
MPA use	0.51	0.28-0.92	0.03	10 (711)	19%	0.18	0.30
Prednisolone use	0.51	0.28-0.94	0.03	8 (637)	0%	0.55	0.71
Graft loss	1.67	0.98-2.86	0.06	11 (780)	21%	0.22	0.009

Abbreviations: Cr, creatinine; HLA, human leukocyte antigen; KT, kidney transplantation; KTR, kidney transplant recipient; MN, membranous nephropathy; MPA, mycophenolic acid; N/A, not available; PLA2R, phospholipase A2 receptor.

a Standardized mean difference (SMD) was used when analyzing the difference between pre-KT anti-PLA2R titer.

**Figure 4:**
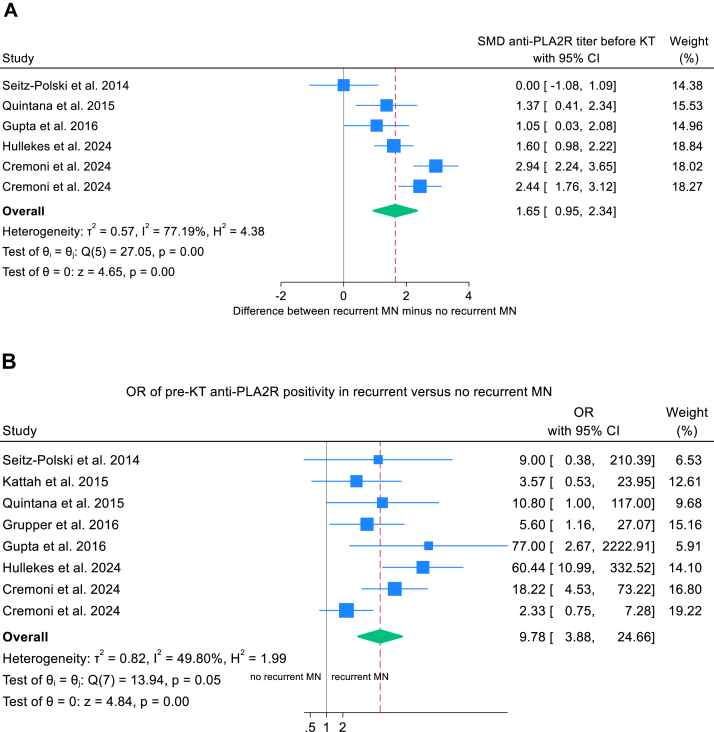
Forest plots of pretransplant anti-PLA2R and recurrent MN. (A) SMD in pretransplant anti-PLA2R titer. (B) Pooled OR for pretransplant anti-PLA2R positivity. CI, confidence interval; KT, kidney transplantation; MN, membranous nephropathy; OR; odds ratio, PLA2R; phospholipase A2 receptor, SMD; standardized mean difference.

Pretransplant anti-PLA2R titers were significantly higher among recipients who experienced recurrent MN compared with those without recurrence (standardized mean difference 1.65; 95% CI, 0.95-2.34; *P* = 0.001). Similarly, the odds of anti-PLA2R positivity were markedly greater in the recurrent MN group (OR, 9.79; 95% CI, 3.88-24.66; *P* < 0.001) (Table 3; Fig 4A and B). Funnel plot asymmetry was observed for anti-PLA2R titers (Egger’s test *P* = 0.008) but not for anti-PLA2R positivity (Egger’s test *P* = 0.30) (Fig S4A and B).

**Figure 5:**
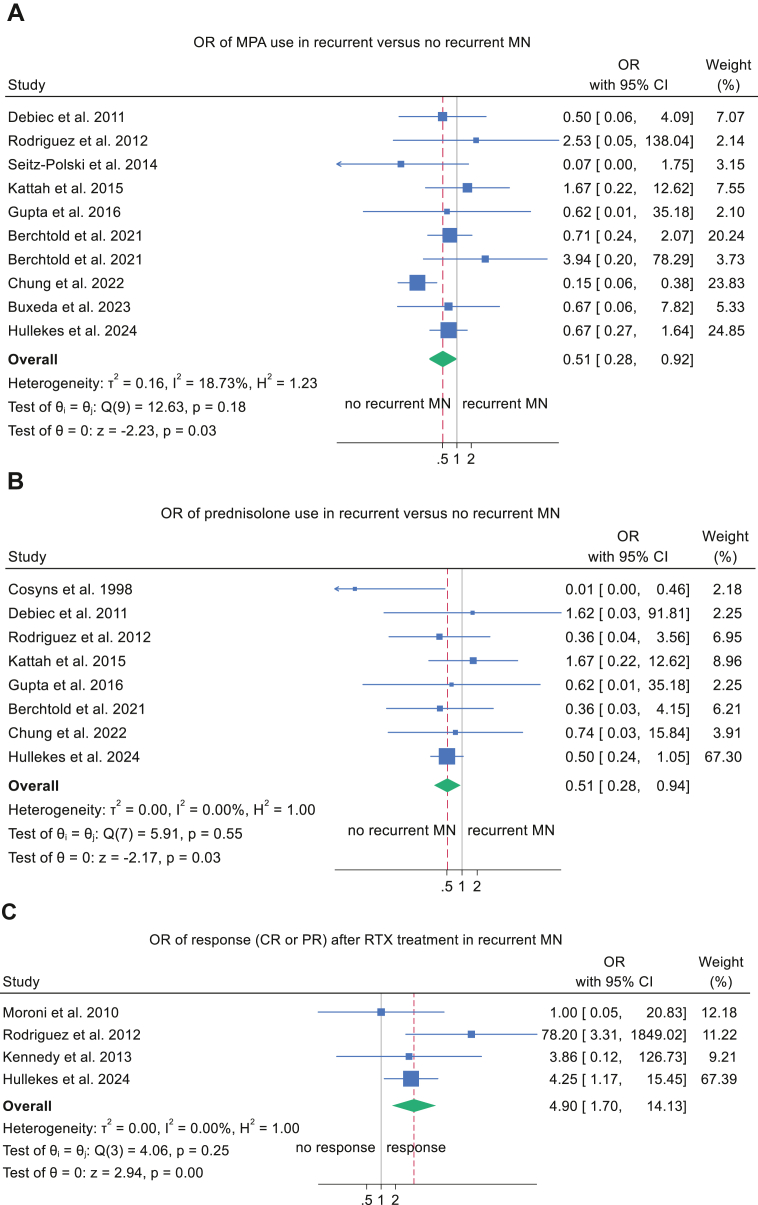
Forest plots of immunosuppressive medications and recurrent MN. (A) Pooled OR for MPA use in recurrent MN. (B) Pooled OR for prednisolone use in recurrent MN. (C) Pooled OR for response after treatment with rituximab in KTR with recurrent nephropathy. CI, confidence interval; CR, complete remission; KTR, kidney transplant recipient; MPA, mycophenolic acid; MN, membranous nephropathy; OR, odds ratio; PR, partial remission.

Use of mycophenolic acid and maintenance corticosteroids was associated with a reduced risk of recurrent MN. Mycophenolic acid decreased the odds of recurrence by 49% (OR, 0.51; 95% CI, 0.28-0.92; *P* = 0.026) (Fig 5A), and maintenance corticosteroid therapy yielded a similar reduction (OR, 0.51; 95% CI, 0.28-0.94; *P* = 0.030) (Fig 5B). Both analyses demonstrated low heterogeneity (*I*^2^ <20 %) and symmetrical funnel plots (Fig S5A and B).

### Meta-analysis of Factors Associated With Recurrent MN Compared With De Novo MN

Table S4 summarizes the meta-analyses of factors associated with recurrent MN versus de novo MN. Recipients with recurrent MN were older (WMD, 11.5 years; 95% CI, 2.0-17.3; *P* < 0.001) and exhibited higher serum creatinine at diagnosis (WMD, 0.74 mg/dL; 95% CI, 0.26-1.22; *P* = 0.003). Forest plots and funnel plots are presented in Figs S6A-D and S7A-D.

Concurrent allograft pathology in de novo MN cases demonstrated greater odds of any rejection compared with recurrent MN (OR, 2.30; 95% CI, 1.16-4.58; P = 0.018) (Figures S8A–B).

### Meta-analysis of Treatment Response After Rituximab Therapy in KTRs With Recurrent and De Novo MN

Four studies^8^^,^^28^^,^^32^^,^^33^ reported sufficient data on 84 KTRs with recurrent MN to permit a meta-analysis of rituximab versus non-rituximab treatment (Fig 5C and Fig S5C). Rituximab use was associated with significantly higher odds of complete or partial remission (OR, 4.90; 95% CI, 1.70-14.13; *P* = 0.003) with no observed heterogeneity (*I*^2^ = 0%; Q-test *P* = 0.25) and no evidence of small-study effects (Egger’s test *P* = 0.82). Mean daily proteinuria decreased from 3,866 ± 4,723 mg pre-rituximab to 1,089 ± 1,929 mg post-rituximab.

No studies of de novo MN provided sufficient data for a comparable meta-analysis, although available reports indicate a reduction in mean proteinuria from 9,118 ± 7,360 mg/d to 670 ± 786 mg/d following rituximab therapy.

### Meta-analysis of Graft Loss in Recurrent Versus Nonrecurrent MN

**Figure 6:**
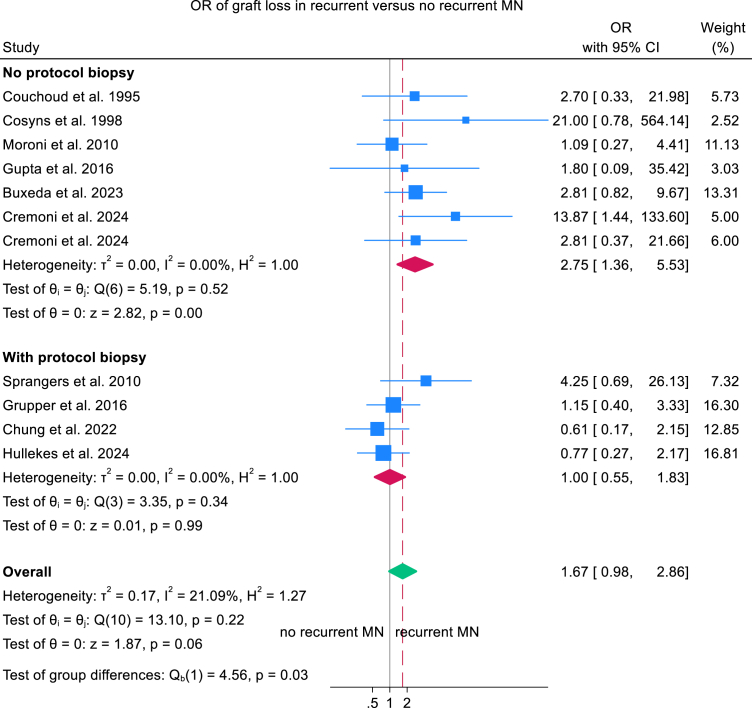
Forest plots comparing recurrent MN with nonrecurrent MN for allograft loss stratified by biopsy indication: protocol biopsies versus no protocol biopsies. CI, confidence interval; MN, membranous nephropathy; OR, odds ratio.

Meta-analyses showed no significant difference in graft loss between KTRs with recurrent MN and those without recurrence (OR, 1.67; 95% CI, 0.98-2.86; *P* = 0.06). However, subgroup analyses stratified by biopsy practice revealed heterogeneity: in centers without protocol biopsies, recurrent MN was associated with higher graft-loss risk than nonrecurrence (OR, 2.75; 95% CI, 1.36-5.53; *P* = 0.005), whereas in centers employing protocol biopsies the association was not significant (between-group difference *P* = 0.033; Fig 6 and Fig S9). Graft loss also did not differ between recurrent and de novo MN (OR, 0.49; 95% CI, 0.19-1.27; *P* = 0.14; Fig S7E and F).

### Meta-regression of Publication Year Versus Allograft Loss in Recurrent MN

**Figure 7:**
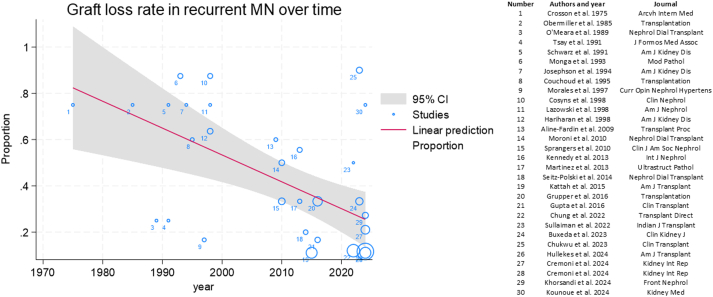
Meta-regression of publication year versus allograft loss rate following recurrent MN. Bubble size is proportional to the number of kidney transplant recipients included in each study. CI, confidence interval; MN, membranous nephropathy.

A meta-regression was conducted to evaluate temporal trends in allograft loss following recurrent MN by assessing the relationship between reported graft loss rates and publication year. The resulting bubble plot demonstrated a significant inverse correlation between publication year and graft loss rate (slope −0.012 per year; *P* = 0.001) (Fig 7).

## Discussion

Recurrent glomerulonephritis after kidney transplantation remains a significant complication, highlighting the need for more robust guidance and evidence in classification, diagnosis, treatment, and, above all, prevention. Among these diseases, MN is characterized by a delayed clinical onset (ie, >6 months posttransplantation) and a potential negative impact on graft survival.^111^^,^^112^ This systematic review and meta-analysis integrates the largest body of evidence to date, including cohort studies, case series, and case reports, and demonstrates an overall MN recurrence rate of approximately one-third. Notably, prevalence was significantly higher in centers performing protocol surveillance biopsies, underscoring the value of routine biopsy protocols for early detection and management of MN in recipients with native kidney failure due to MN.

Meta-analyses identified several demographic factors associated with an increased risk of recurrent MN. Advanced age at transplantation was linked to higher recurrence rates, potentially reflecting age-related immunosenescence and inflammaging, which impair immune regulation.^113^ The interval from native MN diagnosis to kidney failure was significantly shorter among recipients with recurrent MN, a pattern also observed in primary focal segmental glomerulosclerosis recurrence.^114^ Although the mechanism remains unclear, this finding suggests that highly virulent circulating factors driving rapid progression to kidney failure in native disease may persist after transplantation and promote recurrence. Conversely, longer pretransplant dialysis vintage appeared protective against recurrence, possibly due to dialysis-associated immune dysfunction, including autoimmunity, in the chronic uremic state.^115^ This association may be confounded by the tendency for shorter dialysis vintage among living-donor transplant recipients, as discussed below.

Living-donor transplantation was associated with approximately 50% higher risk of recurrent MN in this meta-analysis, with low between-study heterogeneity. This association may reflect genetic polymorphisms in the HLA region (chromosome 6p21) and in the *PLA2R* locus (chromosome 2q24), representing contributions to alloimmunity and autoimmunity, respectively.^116^^,^^117^ Specific HLA-DQA1 and HLA-DRB1 epitopes have been linked to primary MN in nontransplant cohorts, potentially owing to enhanced binding of these epitopes to the PLA2R antigen, which facilitates HLA–peptide complex formation and presentation to T-cell receptors, thereby triggering anti-PLA2R antibody production.^118^ An alternative hypothesis invokes molecular mimicry by certain HLA molecules themselves, independent of bound peptides, as a stimulus for autoantibody generation. Moreover, particular PLA2R epitopes appear especially prone to autoantibody targeting.^119^^,^^120^ Although several studies have explored associations between specific HLA alleles and recurrent MN,^5^^,^^18^^,^^61^ their findings have been heterogeneous and insufficient for pooled analysis, underscoring the need for larger, multicenter investigations. Despite the increased risk of recurrence, living-donor transplantation continues to confer superior graft survival compared with deceased-donor transplantation in recipients with native MN.^121^

Anti-PLA2R antibodies have been established as sensitive biomarkers for the diagnosis, prognosis, and treatment response in primary MN.^122^ This meta-analysis confirms a strong association between recurrent MN and pretransplant anti-PLA2R status: recipients who are seropositive exhibit a 9.8-fold increased risk of recurrence compared with seronegative recipients, and higher antibody titers correlate with greater recurrence risk. However, anti-PLA2R is not pathognomonic: 28.8% of recipients with recurrent MN were seronegative before transplantation, and 23.7% of recipients without recurrence were seropositive (Table 2). Therefore, anti-PLA2R positivity at transplantation should not preclude transplantation, nor should seronegativity be taken as definitive evidence against recurrence. Combining protocol surveillance biopsies with serial posttransplant anti-PLA2R monitoring in recipients whose kidney failure resulted from primary MN may facilitate earlier detection and intervention to prevent graft loss. Reported cutoffs for anti-PLA2R positivity range from 30 to 82 RU/mL, with varying sensitivity and specificity^36^^,^^40^; a universally accepted threshold is not yet defined. The Kidney Disease Improving Global Outcomes (KDIGO) 2021 Clinical Practice Guideline for the Management of Glomerular Diseases mentions a cutoff of >45 RU/mL as indicative of high risk for recurrent MN.^122^ To date, high-quality evidence is lacking to support pretransplant interventions aimed at lowering persistent anti-PLA2R levels to enable safe transplantation and prevent MN recurrence. However, a single report described successful suppression of pretransplant anti-PLA2R with cyclophosphamide, combined with rituximab and intravenous immunoglobulin desensitization and antithymocyte globulin induction, resulting in good allograft function without recurrence at 2 years post-transplant.^123^

De novo MN after kidney transplantation appears to arise via mechanisms distinct from those of recurrent disease. Emerging evidence suggests a stronger association with allograft rejection, particularly antibody-mediated rejection.^104^ Given the heterogeneity in rejection definitions and pathological reporting across studies, all reported instances of rejection were combined as a single outcome in the meta-analysis. The pooled estimate indicated that de novo MN was associated with a 2.3-fold higher odds of allograft rejection compared with recurrent MN; this finding was primarily driven by the cohort described by Batal et al,^61^ despite an *I*^2^ of 0% (Fig S8A; Table S2). Recent studies have identified exostosin and protocadherin FAT1 as putative target antigens in de novo posttransplant MN.^62^^,^^124^ Both antigens were detected alongside antibody-mediated, T cell–mediated, or mixed rejection patterns in biopsy specimens. However, because the number of recipients with de novo MN was limited in this meta-analysis (186 KTRs from 8 studies; Table S4), this result should be interpreted with caution, as a single study may have driven the association. Further investigation is needed to elucidate whether rejection exposes cryptic podocyte antigens and thereby drives autoimmunity or whether alloantibodies against donor-derived podocyte antigen epitopes initiate de novo MN and subsequent allograft rejection.

Maintenance therapy with mycophenolic acid and prednisolone was associated with a reduced risk of recurrent MN, consistent with prior evidence linking steroid withdrawal to recurrence of glomerular diseases.^125^ Mycophenolic acid, a noncompetitive inhibitor of inosine monophosphate dehydrogenase,^126^ selectively impairs de novo purine synthesis required for lymphocyte proliferation and may thereby prevent antibody formation against MN-specific antigens.^127^

Studies from centers without protocol biopsy showed a higher risk of graft loss in recurrent MN, whereas centers with protocol biopsy showed no difference in graft loss between recurrent and nonrecurrent cases. This pattern likely reflects earlier detection at centers with routine surveillance, enabling timelier intervention, while the absence of protocol biopsies may delay diagnosis until allograft injury has already occurred, reducing response to treatment. Additionally, meta-regression demonstrated a significant decline in allograft loss rates among those with recurrent MN over time, a trend likely driven by the increasing adoption of rituximab in recent years. Nonetheless, this improvement may also reflect advances in overall posttransplant care rather than MN-specific therapy alone. Meta-analysis revealed a 4.9-fold increase in the odds of clinical response, defined as complete or partial remission, among rituximab-treated patients compared with non-rituximab regimens. Given the rarity of recurrent MN, randomized controlled trials of rituximab are unlikely without large, multicenter collaborations. On the basis of current evidence, rituximab should be considered first-line therapy for recurrent MN after kidney transplantation.

This systematic review and meta-analysis offers several strengths. It is the first study to comprehensively evaluate all forms of posttransplant MN—recurrent, nonrecurrent, and de novo—across prevalence, risk factors, clinical associations, treatment responses, and graft outcomes using a uniform, protocol-driven literature search. Although some factors have been reported in the literature, their effect sizes have not been consistently quantified across studies. The objective of this meta-analysis was therefore to include all available evidence and derive the most up-to-date, aggregate estimates of effect. The finding that protocol biopsies are linked to higher detection rates of recurrent MN highlights the potential value of surveillance biopsies in recipients whose kidney failure was caused by MN. In addition to confirming established risk factors such as pretransplant anti-PLA2R positivity and living-donor transplantation, this analysis identifies novel predictors of recurrence, including shorter dialysis vintage, a shorter interval from diagnosis to kidney failure, and maintenance therapy without mycophenolic acid and prednisolone. Finally, the demonstrated efficacy of rituximab and the observed decline in graft-loss rates over time provide new insights into optimal management strategies for posttransplant MN.

Several limitations warrant consideration. First, only circulating anti-PLA2R antibody levels could be assessed in this meta-analysis due to data availability. Alternative prognostic markers, such as PLA2R antigen staining in native kidney tissue or antibodies against thrombospondin type-1 domain-containing protein 7A, could not be evaluated because corresponding data remain scarce.^34^^,^^107^ In addition, evidence on the stage or severity of posttransplant MN and its association with allograft outcomes is lacking. Second, the contribution of specific HLA and PLA2R epitopes to recurrent or de novo MN could not be quantified, as reporting was both limited and inconsistent across studies. Third, no sufficient evidence was available to make recommendations on preemptive rituximab induction in transplant candidates with native MN. Fourth, substantial heterogeneity was observed for several factors, including dialysis vintage, interval from native MN diagnosis to kidney failure, anti-PLA2R titer, and recurrence prevalence, necessitating cautious interpretation. The pooled estimates for these variables should be considered hypothesis-generating. The high heterogeneity likely reflects differences in case mix, definitions (eg, dialysis vintage/time anchors), and assay methodologies. Evidence of funnel-plot asymmetry (significant Egger’s tests) indicates small-study effects or publication bias for age, pretransplant anti-PLA2R antibody titer, and graft loss. Consequently, pooled effect sizes may be overestimated. Larger, harmonized registries with standardized definitions are needed to yield more precise effect-size estimates. Finally, all available studies identified in the electronic databases were included. Although this approach minimizes selection bias, it can introduce substantial heterogeneity across eras of clinical practice. To aid interpretation, forest plots for each meta-analysis are presented with studies arranged chronologically to visualize potential temporal trends. In addition, meta-regression was performed to relate outcomes to year of publication and to summarize contemporary improvements in the care of KTRs with MN.

In conclusion, this systematic review and meta-analysis delivers a comprehensive estimate of posttransplant recurrent MN prevalence. The findings support adopting protocol surveillance biopsies in recipients with a history of native MN. Previously described risk factors have been confirmed, and novel predictors have been identified. Among available therapies, rituximab has the strongest supporting evidence and should be considered first-line for recurrent MN.
