# Supplementary material for: Recurrent, Nonrecurrent, and De Novo Membranous Nephropathy After Kidney Transplantation: A Systematic Review and Meta-Analysis
Source: Kidney Med. 2026 Feb 6;8(4):101284. doi: 10.1016/j.xkme.2026.101284 (PMC13019562; doi:10.1016/j.xkme.2026.101284)
Supplement: Supplementary File (PDF) — Table S1-S4; Figure S1-S8. [file mmc1.pdf]

Recurrent, Non-Recurrent, and De Novo Membranous Nephropathy After Kidney Transplantation: A Systemic Review and Meta-Analysis

Thanyarat Phumthian, MD[1,2], Veerapat Wattanasatja, MD[1,3], Aschariya Wipattanakitcharoen, MD[1], Thunyatorn Wuttiputhanun, MD[1,4], Asada Leelahavanichkul, MD, PhD[5,6], Natavudh Townamchai, MD[1,4,7], Yingyos Avihingsanon, MD[1,4,7], Suwasin Udomkarnjananun, MD, PhD[1,4,5,7]

Authors' Affiliations:

[1]Division of Nephrology, Department of Medicine, Faculty of Medicine, Chulalongkorn University and King Chulalongkorn Memorial Hospital, The Thai Red Cross Society, Bangkok, Thailand

[2]Department of Internal Medicine, Ubon Ratchathani University Hospital; College of Medicine and Public Health, Ubon Ratchathani University, Ubon Ratchathani, Thailand

[3]Department of Internal Medicine, Sunpasitthiprasong Hospital, Ubon Ratchathani, Thailand

[4]Excellence Center for Organ Transplantation (ECOT), King Chulalongkorn Memorial Hospital, The Thai Red Cross Society, Bangkok, Thailand.

[5]Center of Excellence on Translational Research in Inflammation and Immunology (CETRII), Department of Microbiology, Faculty of Medicine, Chulalongkorn University, Bangkok, Thailand.

[6]Immunology Unit, Department of Microbiology, Chulalongkorn University, Bangkok, Thailand.

[7]Renal Immunology and Renal Transplant Center of Excellence, Faculty of Medicine, Chulalongkorn University, Bangkok, Thailand.

Table S1: Case series and case reports included in the systematic review and meta-analysis

Case series								
**Author**	**Year of publication**	**Journal**	**Country**	**Recurrent MN**	**De novo MN**	**Protocol biopsy**	**Total patients included**	**Treatment with Rituximab**
Steinmuller et al. (47)	1978	Clin Nephrol	USA	No	Yes	No	2	No
Cosyns et al. (48)	1982	Kidney Int	Belgium	No	Yes	No	9	No
Truong et al. (49)	1989	Am J Kidney Dis	USA	No	Yes	No	10	No
Tsay et al. (50)	1991	J Formos Med Assoc	Taiwan	Yes	Yes	No	5	No
Monga et al. (51)	1993	Mod Pathol	Italy	Yes	Yes	No	18	No
Schwarz et al. (52)	1994	Transplantation	Germany	No	Yes	No	21	No
Marcen et al. (53)	1996	Nephrol Dial Transplant	Spain	Yes	No	No	3	No
Cosyns et al. (5)	1998	Clin Nephrol	Belgium and France	Yes	No	No	42	No
Kearney et al. (54)	2011	Transplant Proc	USA	Yes	Yes	No	9	No
Andrésdóttir et al. (55)	2012	Am J Transplant	North America	Yes	No	No	23	No
Martínez et al. (56)	2013	Ultrastruct Pathol	Spain	Yes	No	No	6	No
Seitz-Polski et al. (57)	2014	Nephrol Dial Transplant	France	Yes	No	Yes	15	No
Kattah et al. (58)	2016	Am J Transplant	USA	Yes	No	Yes	18	No
Cosio et al. (59)	2017	Kidney Int	North America	Yes	No	Yes	49	No
Katsuma et al. (60)	2018	Nephrology	Japan	No	Yes	No	5	No

Batal et al. (61)	2020	Am J Kidney Dis	USA, Canada, France	Yes	Yes	Yes	77	No
Al-Rabadi et al. (62)	2025	Kidney Int Rep	USA	No	Yes	Yes	6	No
**Case reports**								
**Author**	**Year of publication**	**Journal**	**Country**	**Recurrent MN**	**De novo MN**	**Protocol biopsy**	**Total patients included**	**Treatment with Rituximab**
Crosson et al. (63)	1975	Arcvh Intern Med	USA	Yes	No	No	1	No
Rubin et al. (64)	1977	Transplantation	USA	Yes	No	No	1	No
Lieberthal et al. (65)	1979	Clin Nephrol	USA	Yes	No	No	1	No
Grizzle et al. (66)	1981	Arch Pathol Lab Med	USA	No	Yes	No	1	No
Sugisaki et al. (67)	1982	Transplantation	USA	No	Yes	No	1	No
Obermiller et al. (68)	1985	Transplantation	USA	Yes	No	No	1	No
Ahmad et al. (69)	1992	J Pak Med Assoc	Pakistan	No	Yes	No	1	No
Agarwal et al. (70)	1992	Nephron	India	Yes	No	No	1	No
Innes et al. (71)	1994	Nephrol Dial Transplant	UK	Yes	No	No	1	No
Josephson et al. (72)	1994	Am J Kidney Dis	USA	Yes	No	No	1	No
Song et al. (73)	1996	J Korean Med Sci	Korea	No	Yes	No	1	No
Lal et al. (74)	1997	Int J Artif Organs	USA	No	Yes	No	1	No
Lazowski et al. (75)	1998	Am J Nephrol	USA	Yes	No	No	1	No
Ideura et al. (76)	2000	Transplant Proc	Japan	No	Yes	No	1	No
Miserlis et al. (77)	2002	Hippokratia	Greece	No	Yes	No	1	No
Teixeira et al. (78)	2002	Transplant Proc	Portugal	No	Yes	No	1	No

Ashfaq et al. (79)	2004	J Nephrol	USA	Yes	No	No	1	No
Gallon et al. (80)	2006	Am J Transplant	USA	Yes	No	No	1	Yes
Carrasco et al. (81)	2008	Transplant Proc	USA	Yes	No	No	1	No
El Kossi et al. (82)	2008	Clin Transplant	UK	No	Yes	No	1	No
Liebl et al. (83)	2008	Clin Nephrol	Germany	Yes	No	No	1	No
Sirimongkolrat et al. (84)	2008	Transplant Proc	Thailand	No	Yes	No	1	No
Weclawiak et al. (85)	2008	Clin Nephrol	France	Yes	No	No	1	Yes
Ladino et al. (86)	2009	Nephrol Dial Transplant Plus	USA	Yes	No	No	1	Yes
Kfoury et al. (87)	2010	Transplant Proc	Saudi Arabia	No	Yes	No	1	No
Stahl et al. (88)	2010	N Engl J Med	Germany	Yes	No	No	1	Yes
Damodar et al. (89)	2011	Clinical Transplantation	USA	Yes	No	No	1	Yes
Blosser et al. (90)	2012	Am J Transplant	USA	Yes	No	No	1	No
Debiec et al. (91)	2012	J Am Soc Nephrol	France	Yes	No	No	1	Yes
Hinkes et al. (92)	2012	BMC Nephrol	Germany	No	Yes	No	1	No
Okyay et al. (93)	2012	Ren Fail	Turkey	No	Yes	No	1	No
Taton et al. (94)	2013	Transpl Infect Dis	France	No	Yes	No	1	No
Makhdoomi et al. (95)	2015	Iran J Kidney Dis	Iran	Yes	No	No	1	Yes
Saito et al. (96)	2015	Nephrology	Japan	No	Yes	No	1	No
Doke et al. (97)	2016	Internal Medicine	Japan	No	Yes	No	1	No
Barbari et al. (98)	2017	Exp Clin Transplant	Lebanon	Yes	No	No	1	Yes
Ishiwatari et al. (99)	2018	Transplant Proc	Japan	Yes	No	No	1	Yes
Kumar et al. (100)	2018	Saudi J Kidney Dis Transpl	India	Yes	No	No	1	No

Murakami et al. (101)	2018	Transpl Infect Dis	USA	Yes	No	No	1	Yes
Steyaert et al. (102)	2018	Acta Clin Belg	Belgium	Yes	No	Yes	1	No
Münch et al. (103)	2021	Am J Transplant	Germany	No	Yes	No	1	No
Darji et al. (104)	2022	World J Transplant	India	No	Yes	No	1	No
Giannopoulou et al. (105)	2022	Exp Clin Transplant	Greece	Yes	No	No	1	Yes
Sullaiman et al. (106)	2022	Indian J Transplant	India	Yes	No	No	1	No
Solà-Porta et al. (107)	2023	Nefrologia	Spain	No	Yes	Yes	1	No
Fukuda et al. (108)	2024	CEN Case Rep	Japan	No	Yes	No	1	No
Kounoue et al. (109)	2024	Kidney Med	Japan	Yes	No	No	1	No
Sridharan et al. (110)	2025	J Nephrol	Australia	Yes	No	No	1	Yes

Table S2: Quality assessment of cohort studies using the Newcastle-Ottawa Scale (NOS).

Author	Year of publication	Selection (4)				Comparability (2)	Outcome (3)			Total (9)
		Case Definition (1)	Selection of the non-exposed cohort: (1)	Ascertainment (1)	Demonstration that outcome of interest was not present at start of study (1)		Assessment of outcome: (1)	Was follow-up long enough (1)	Adequacy of follow up of cohorts: (1)	
Morzycka et al. (13)	1982	1	1	1	1	0	1	1	1	7
Honkanen et al. (14)	1984	1	1	1	1	0	1	1	1	7
Ward et al. (15)	1988	1	1	1	1	0	1	0	0	5
O'Meara et al. (16)	1989	1	1	1	1	0	1	1	1	8
Schwarz et al. (17)	1991	1	0	0	1	0	1	1	0	4
Couchoud et al. (18)	1995	0	1	1	1	0	1	0	1	5
Odorico et al. (19)	1996	1	1	1	1	1	1	1	1	8
Morales et al. (20)	1997	1	1	1	1	0	1	1	1	7
Hariharan et al. (21)	1998	1	1	1	1	0	1	1	1	7
Hariharan et al. (22)	1999	1	1	1	1	1	1	1	1	8
Briganti et al. (23)	2002	1	1	1	1	2	1	1	1	9
Ibrahim et al. (24)	2006	1	1	1	1	1	1	1	1	8
Dabade et al. (25)	2008	1	1	1	1	0	1	1	1	7
Aline-Fardin et al. (26)	2009	1	1	1	1	0	1	1	0	6

El-Zoghby et al. (27)	2009	1	1	1	1	1	1	1	1	8
Moroni et al. (28)	2010	1	1	1	1	2	1	1	1	9
Sprangers et al. (29)	2010	1	1	1	1	1	1	1	1	8
Debiec et al. (30)	2011	0	0	1	1	0	1	0	1	4
Honda et al. (31)	2011	1	1	1	1	2	1	1	1	9
Rodriguez et al. (32)	2012	0	1	1	1	0	1	1	1	6
Kennedy et al. (33)	2013	1	0	1	1	0	1	1	1	6
Larsen et al. (34)	2013	1	1	1	1	1	1	0	1	7
Kattah et al. (35)	2015	1	1	1	1	0	1	1	1	7
Quintana et al. (36)	2015	1	1	1	1	0	1	1	1	7
Spinner et al. (37)	2015	1	1	1	1	1	1	1	1	8
Wen et al. (38)	2016	1	1	1	1	1	1	1	0	7
Grupper et al. (39)	2016	1	1	1	1	1	1	1	0	7
Gupta et al. (40)	2016	1	1	1	1	0	1	1	1	7
Jiang et al. (41)	2018	1	1	1	1	2	1	1	1	9
Singh et al. (42)	2019	1	1	1	1	2	1	1	1	9
Berchtold et al. (43)	2021	1	1	1	1	2	1	1	0	8
Chung et al. (7)	2022	1	1	1	1	1	1	1	0	7
Buxeda et al. (44)	2023	1	1	1	1	2	1	1	1	9

Chukwu et al. (45)	2023	1	1	1	1	2	1	1	1	9
Cremoni et al. (6)	2024	1	1	1	1	2	1	1	1	9
Hullekes et al. (8)	2024	1	1	1	1	2	1	1	1	9
Khorsandi et al. (46)	2024	1	1	1	1	1	1	1	1	8

Table S3: Joanna Briggs Institute (JBI) Critical Appraisal Checklists for case series and case reports

Case series											
**Author**	**Year of publication**	**Inclusion**	**Standard measure**	**Valid method**	**Consecutive inclusion**	**Completes inclusion**	**Demographics**	**Clinical**	**Results**	**Site**	**Statistical analysis**
Steinmuller et al. (47)	1978	Yes	Yes	Yes	Yes	Yes	Yes	Yes	Yes	Yes	No
Cosyns et al. (48)	1982	Yes	Yes	Yes	No	No	Yes	Yes	Yes	Yes	No
Truong et al. (49)	1989	Yes	Yes	Yes	No	Yes	Yes	Yes	Yes	Yes	No
Tsay et al. (50)	1991	Yes	Yes	Yes	Yes	Yes	Yes	Yes	Yes	Yes	Yes
Monga et al. (51)	1993	Yes	Yes	Yes	Yes	Yes	Yes	Yes	Yes	Yes	Yes
Schwarz et al. (52)	1994	Yes	Yes	Yes	Yes	Yes	Yes	Yes	Yes	Yes	Yes
Marcen et al. (53)	1996	Yes	Yes	Yes	Yes	Yes	Yes	Yes	Yes	Yes	Yes
Cosyns et al. (5)	1998	Yes	Yes	Yes	Yes	Yes	Yes	Yes	Yes	Yes	Yes
Kearney et al. (54)	2011	Yes	Yes	Yes	Yes	Yes	Yes	Yes	Yes	Yes	Yes
Andrésdóttir et al. (55)	2012	Yes	Yes	Yes	Yes	Yes	Yes	Yes	Yes	Yes	Yes
Martínez et al. (56)	2013	Yes	Yes	Yes	Yes	Yes	Yes	Yes	Yes	Yes	Yes
Seitz-Polski et al. (57)	2014	Yes	Yes	Yes	Yes	Yes	Yes	Yes	Yes	Yes	Yes
Kattah et al. (58)	2016	Yes	Yes	Yes	Yes	Yes	Yes	Yes	Yes	Yes	Yes
Cosio FG et al. (59)	2017	Yes	Yes	Yes	Yes	Yes	Yes	Yes	Yes	Yes	Yes
Katsuma et al. (60)	2018	Yes	Yes	Yes	Yes	Yes	Yes	Yes	Yes	Yes	Yes

Batal et al. (61)	2020	Yes	Yes	Yes	Yes	Yes	Yes	Yes	Yes
Al-Rabadi et al. (62)	2025	Yes	Yes	Yes	Yes	Yes	Yes	Yes	Yes

Case reports

Author	Year of publication	Demographic	History and timeline	Clinical condition	Method and result	Intervention	Post-intervention	Adverse events	Lessons
Crosson et al. (63)	1975	Yes	Yes	Yes	Yes	Yes	Yes	Yes	Yes
Rubin et al. (64)	1977	Yes	Yes	Yes	Yes	No	No	No	Yes
Lieberthal et al. (65)	1979	Yes	Yes	Yes	Yes	Yes	Yes	Yes	Yes
Grizzle et al. (66)	1981	Yes	Yes	Yes	Yes	Yes	Yes	Yes	Yes
Sugisaki et al. (67)	1982	Yes	Yes	Yes	Yes	Yes	Yes	Yes	Yes
Obermiller et al. (68)	1985	Yes	Yes	Yes	Yes	Yes	Yes	Yes	Yes
Ahmad et al. (69)	1992	Yes	Yes	Yes	Yes	Yes	Yes	Yes	Yes
Agarwal et al. (70)	1992	Yes	Yes	Yes	Yes	Yes	Yes	Yes	Yes
Innes et al. (71)	1994	Yes	Yes	Yes	Yes	Yes	Yes	Yes	Yes
Josephson et al. (72)	1994	Yes	Yes	Yes	Yes	Yes	Yes	Yes	Yes
Song et al. (73)	1996	Yes	Yes	Yes	Yes	No	No	No	Yes
Lal et al. (74)	1997	Yes	Yes	Yes	Yes	Yes	Yes	No	Yes
Lazowski et al. (75)	1998	Yes	Yes	Yes	Yes	Yes	Yes	Yes	Yes
Ideura et al. (76)	2000	Yes	Yes	Yes	Yes	Yes	Yes	No	Yes
Miserlis et al. (77)	2002	Yes	Yes	Yes	Yes	Yes	Yes	Yes	Yes

Teixeira et al. (78)	2002	Yes	Yes	Yes	Yes	Yes	Yes	No	Yes
Ashfaq et al. (79)	2004	Yes	Yes	Yes	Yes	Yes	Yes	Yes	Yes
Gallon et al. (80)	2006	Yes	Yes	Yes	Yes	Yes	Yes	Yes	Yes
Carrasco et al. (81)	2008	Yes	Yes	Yes	Yes	Yes	Yes	Yes	Yes
El Kossi et al. (82)	2008	Yes	Yes	Yes	Yes	Yes	Yes	Yes	Yes
Liebl et al. (83)	2008	Yes	Yes	Yes	Yes	Yes	Yes	Yes	Yes
Sirimongkolrat et al. (84)	2008	Yes	Yes	Yes	Yes	Yes	Yes	Yes	Yes
Weclawiak et al. (85)	2008	Yes	Yes	Yes	Yes	Yes	Yes	Yes	Yes
Ladino et al. (86)	2009	Yes	Yes	Yes	Yes	Yes	Yes	Yes	Yes
Kfoury et al. (87)	2010	Yes	Yes	Yes	Yes	Yes	Yes	No	Yes
Stahl et al. (88)	2010	Yes	Yes	Yes	Yes	Yes	Yes	Yes	Yes
Damodar et al. (89)	2011	Yes	Yes	Yes	Yes	Yes	Yes	Yes	Yes
Blosser et al. (90)	2012	Yes	Yes	Yes	Yes	Yes	Yes	Yes	Yes
Debiec et al. (91)	2012	Yes	Yes	Yes	Yes	Yes	Yes	Yes	Yes
Hinkes et al. (92)	2012	Yes	Yes	Yes	Yes	Yes	Yes	Yes	Yes
Okyay et al. (93)	2012	Yes	Yes	Yes	Yes	Yes	Yes	Yes	Yes
Taton et al. (94)	2013	Yes	Yes	Yes	Yes	Yes	Yes	No	Yes
Makhdoomi et al. (95)	2015	Yes	Yes	Yes	Yes	Yes	Yes	Yes	Yes
Saito et al. (96)	2015	Yes	Yes	Yes	Yes	Yes	Yes	No	Yes

Doke et al. (97)	2016	Yes	Yes	Yes	Yes	Yes	Yes	No	Yes
Barbari et al. (98)	2017	Yes	Yes	Yes	Yes	Yes	Yes	Yes	Yes
Ishiwatari et al. (99)	2018	Yes	Yes	Yes	Yes	Yes	Yes	Yes	Yes
Kumar et al. (100)	2018	Yes	Yes	Yes	Yes	Yes	Yes	Yes	Yes
Murakami et al. (101)	2018	Yes	Yes	Yes	Yes	Yes	Yes	Yes	Yes
Steyaert et al. (102)	2018	Yes	Yes	Yes	Yes	Yes	Yes	Yes	Yes
Münch et al. (103)	2021	Yes	Yes	Yes	Yes	Yes	Yes	No	Yes
Darji et al. (104)	2022	Yes	Yes	Yes	Yes	Yes	Yes	No	Yes
Giannopoulou et al. (105)	2022	Yes	Yes	Yes	Yes	Yes	Yes	Yes	Yes
Sullaiman et al. (106)	2022	Yes	Yes	Yes	Yes	Yes	Yes	Yes	Yes
Solà-Porta et al. (107)	2023	Yes	Yes	Yes	Yes	Yes	Yes	Yes	Yes
Fukuda et al. (108)	2024	Yes	Yes	Yes	Yes	Yes	Yes	Yes	Yes
Kounoue et al. (109)	2024	Yes	Yes	Yes	Yes	Yes	Yes	Yes	Yes
Sridharan et al. (110)	2025	Yes	Yes	Yes	Yes	Yes	Yes	Yes	Yes

Table S4: Meta-analysis of factors associated with recurrent MN versus de novo MN in KTR

Variables	Weighted mean difference (WMD)	95%CI	p-value	Studies included (total KTRs included)	I^2 index (%)	Q-test p-value	Egger's test p-value
Age at KT, months	11.5	5.0 to 17.3	<0.001	10 (220)	62	<0.001	0.63
Time from KT to MN, months	-7.2	-37.8 to 23.3	0.64	7 (195)	95	<0.001	0.34
Serum Cr at post-KT MN diagnosis, mg/dL	0.74	0.26 to 1.22	0.003	4 (81)	0	0.42	0.84
Urine protein at post-KT MN diagnosis, mg/day	2,741	-3,597 to 9,078	0.40	5 (84)	93	<0.001	0.57
**Variables**	**Pooled odds ratio (OR)**	**95%CI**	**p-value**	**Studies included (total KTRs included)**	**I^2 index (%)**	**Q-test p-value**	**Egger's test p-value**
Any allograft rejection[a]	2.30	1.16 to 4.58	0.02	8 (186)	0	0.36	0.07
Graft loss	0.49	0.19 to 1.27	0.14	7 (116)	0	0.74	0.51

[a] De novo MN was compared with recurrent MN, which served as the reference group.

Cr; creatinine, KT; kidney transplantation, KTR; kidney transplant recipient, MN; membranous nephropathy

Figure S1: Funnel plot of the meta-analysis of recurrent membranous nephropathy prevalence.

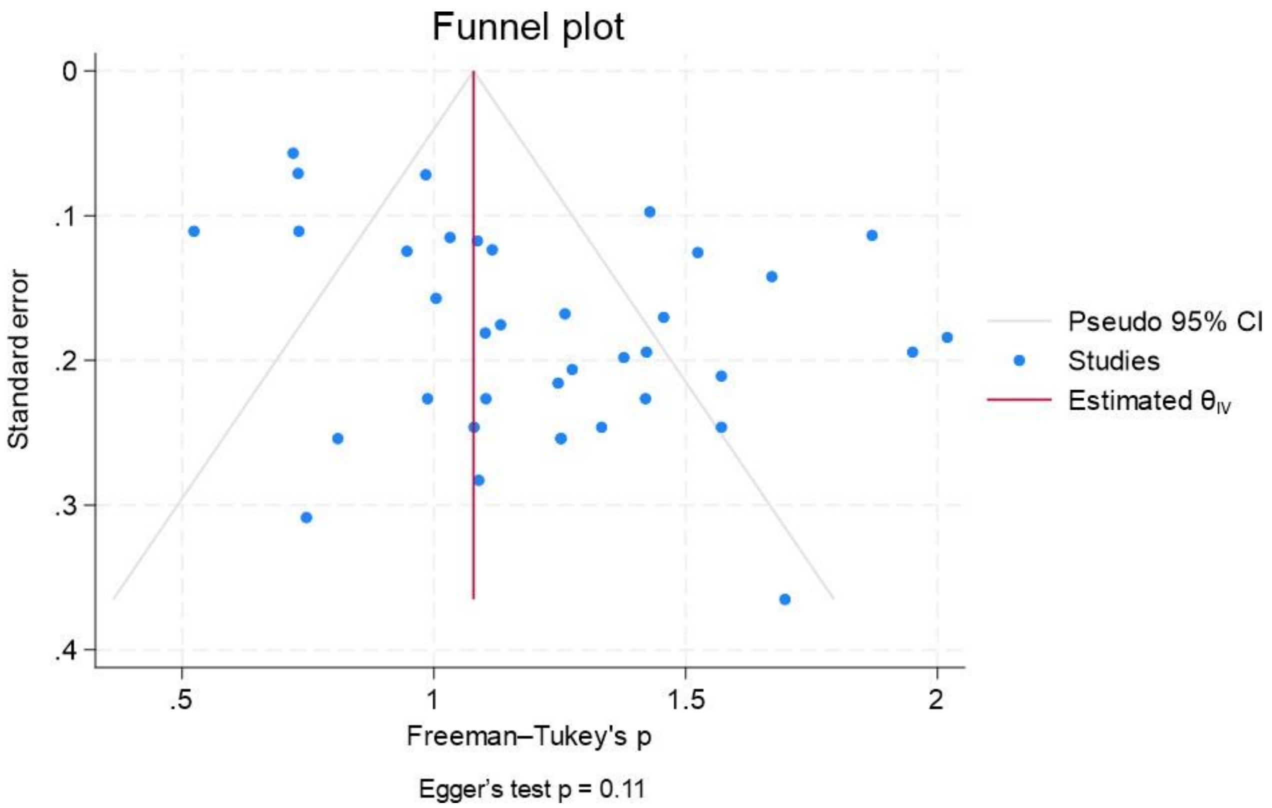

Figure S2: Funnel plots assessing potential publication bias in meta-analyses of baseline characteristics and recurrent membranous nephropathy. (A) Age at transplantation. (B) Dialysis vintage. (C) Interval from native membranous nephropathy to end-stage kidney disease. (D) Living donor kidney transplantation.

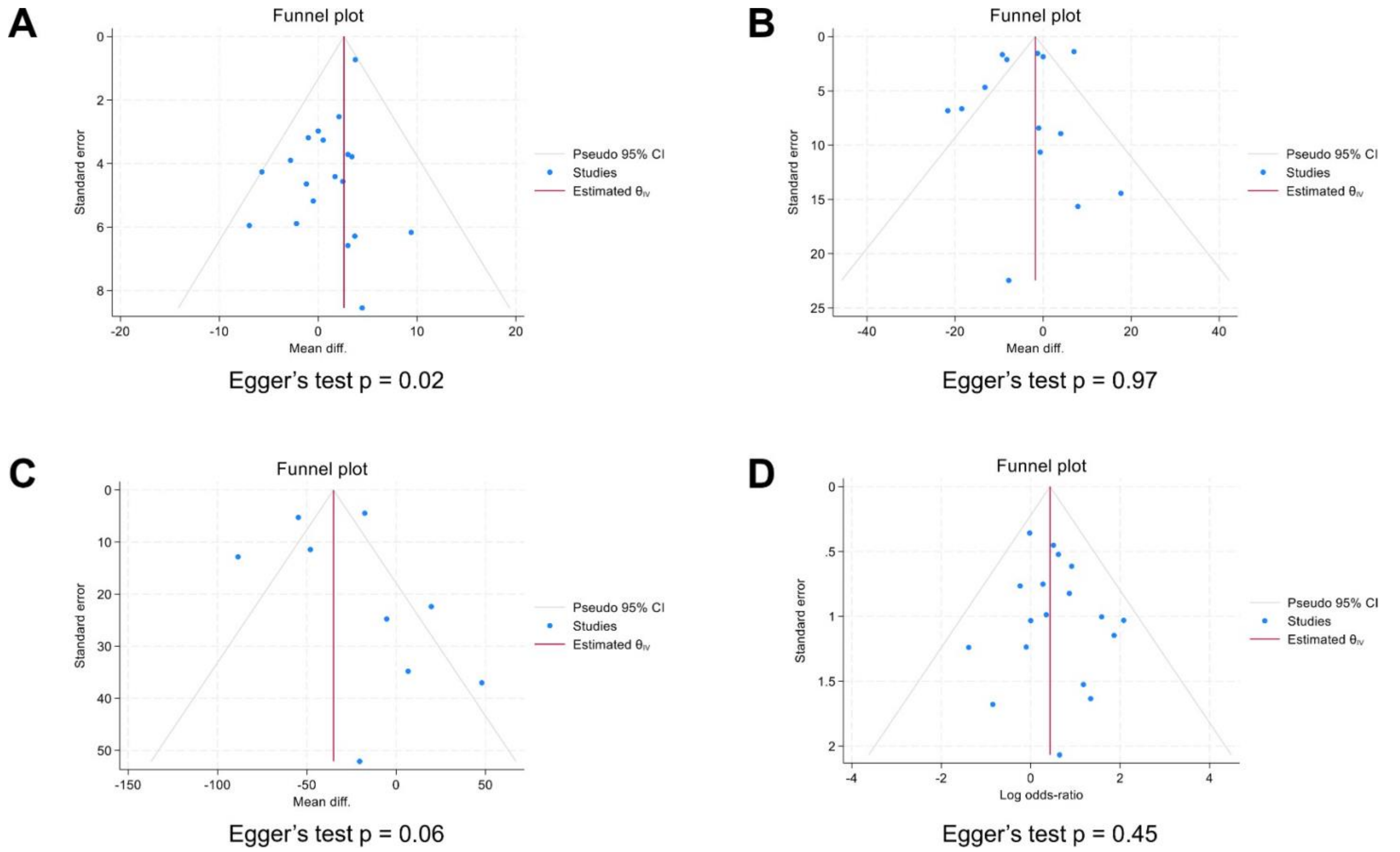

Figure S3: Forest and funnel plots showing the associations between recurrent membranous nephropathy and each variable: male sex (A and B), use of T-cell depleting induction therapy (C and D), and tacrolimus use (E and F).

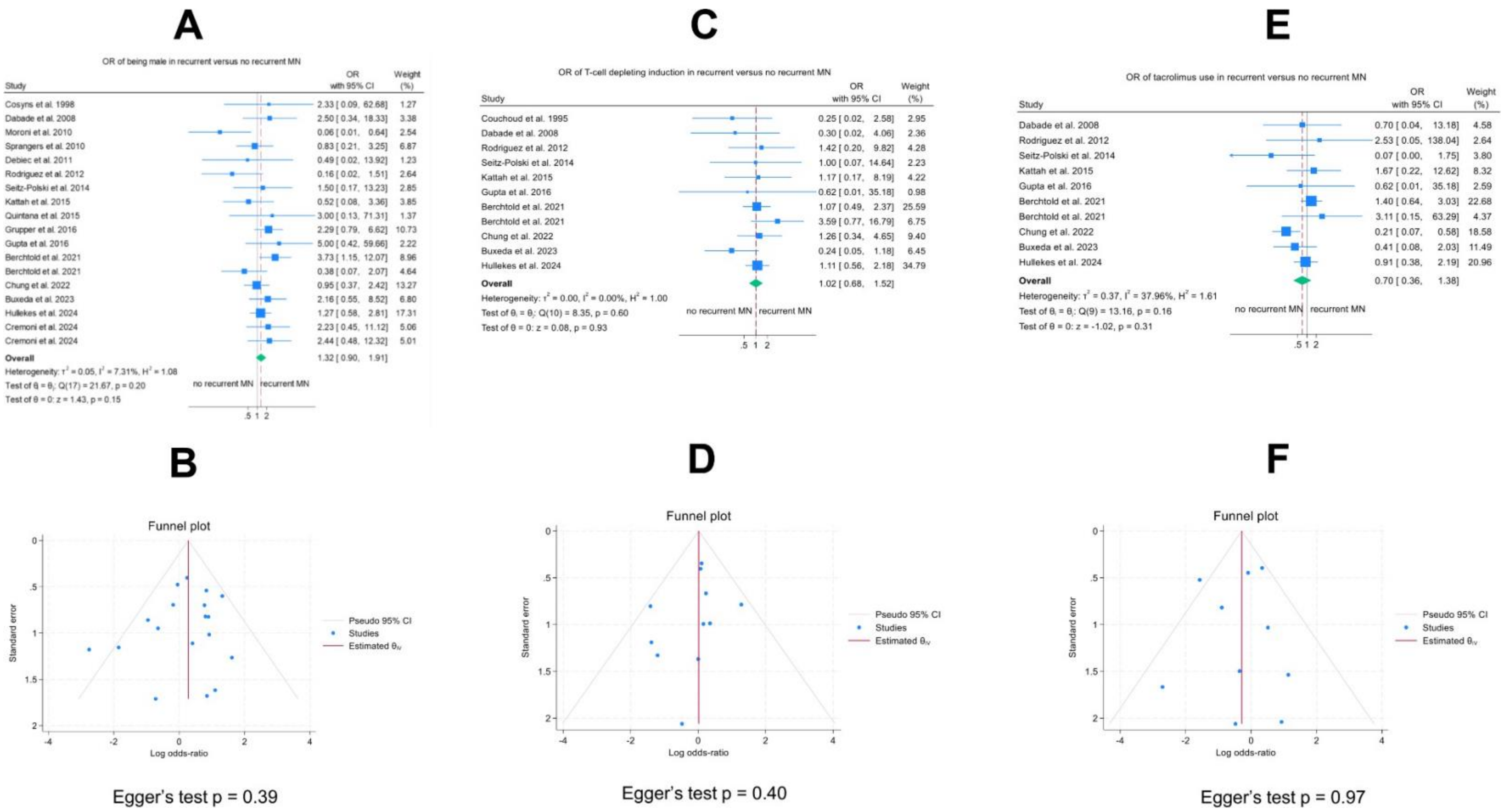

Figure S4: Funnel plots assessing potential publication bias in meta-analyses of anti-PLA_2R and recurrent membranous nephropathy: (A) anti-PLA2R titer. (B) anti-PLA2R positivity. PLA2R, phospholipase A2 receptor.

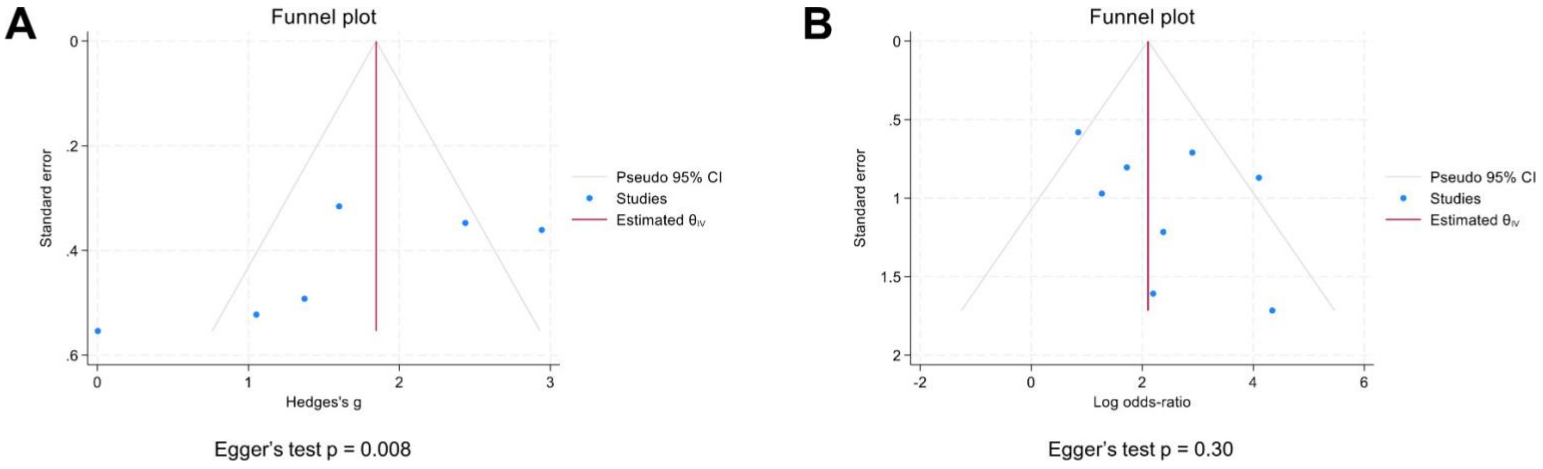

Figure S5: Funnel plots assessing potential publication bias in meta-analyses of immunosuppressive therapies and recurrent membranous nephropathy: (A) Mycophenolic acid use. (B) Prednisolone use. (C) Response to rituximab treatment.

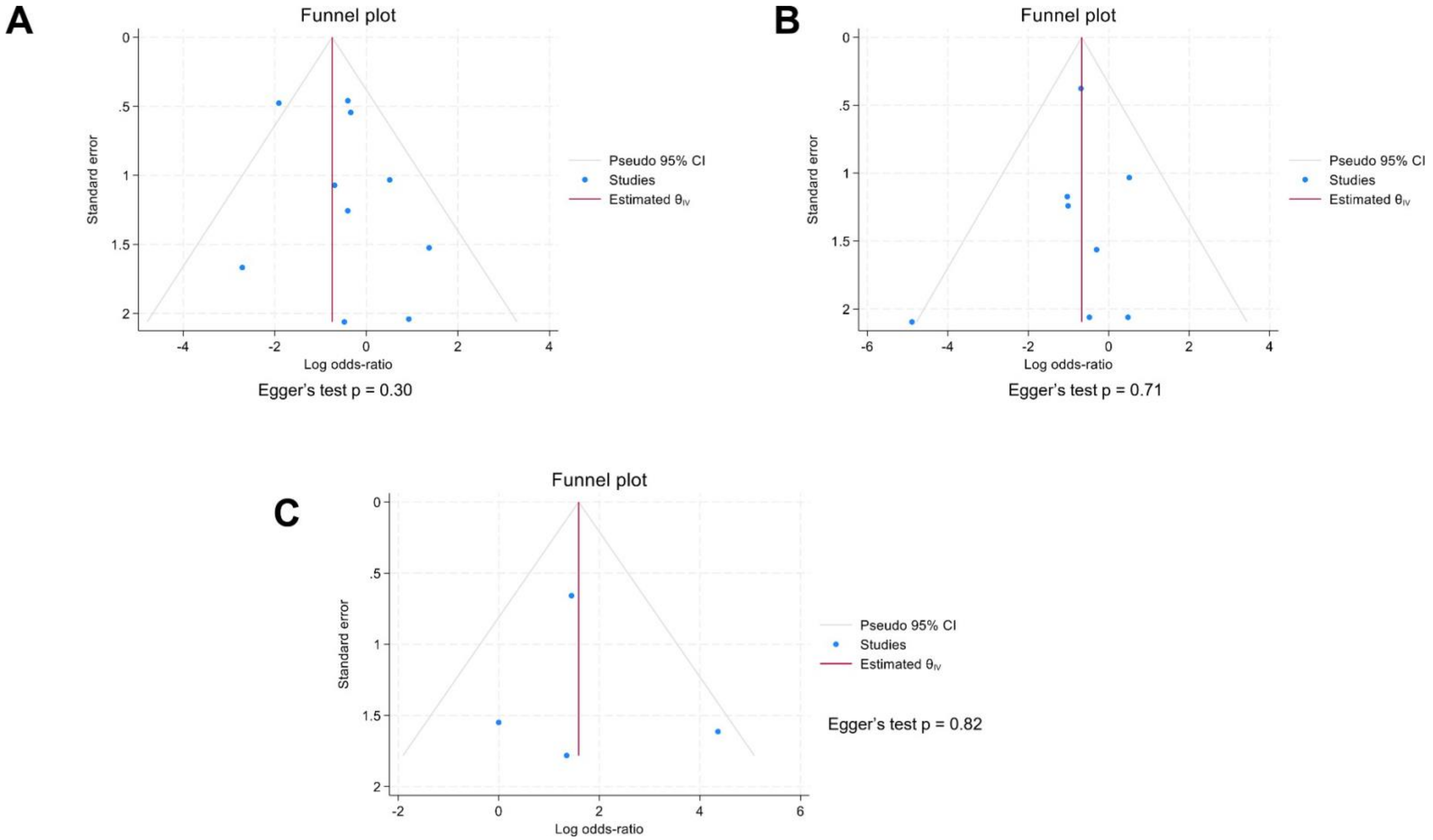

Figure S6: Forest and funnel plots comparing recurrent membranous nephropathy with de novo membranous nephropathy for each variable: Age at transplantation (A and B) and interval from transplantation to membranous nephropathy diagnosis (C and D).

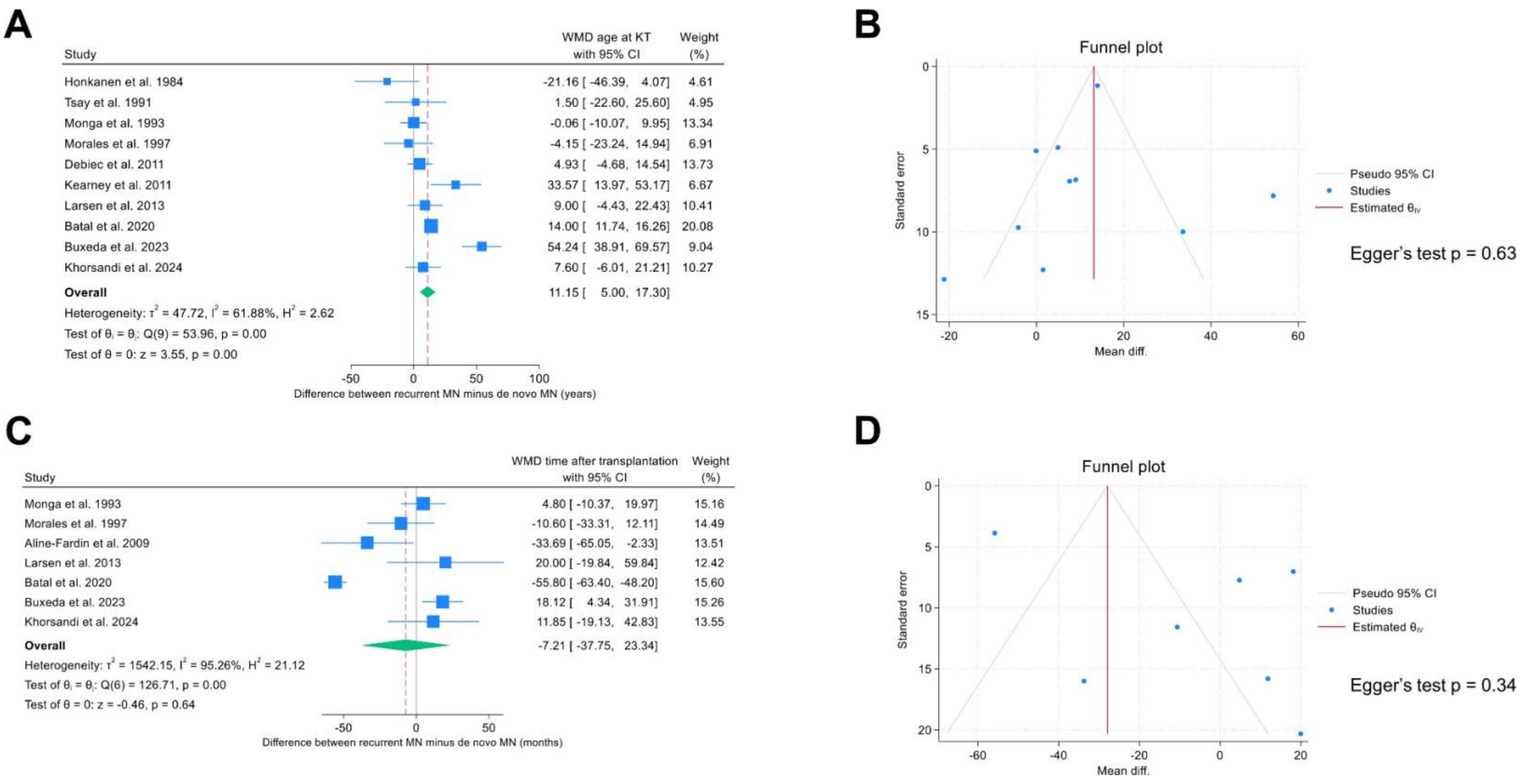

Figure S7: Forest and funnel plots comparing recurrent membranous nephropathy with de novo membranous nephropathy for each variable: Serum creatinine at time of membranous nephropathy diagnosis (A and B), urine protein at time of membranous nephropathy diagnosis (C and D), and kidney allograft loss (E and F).

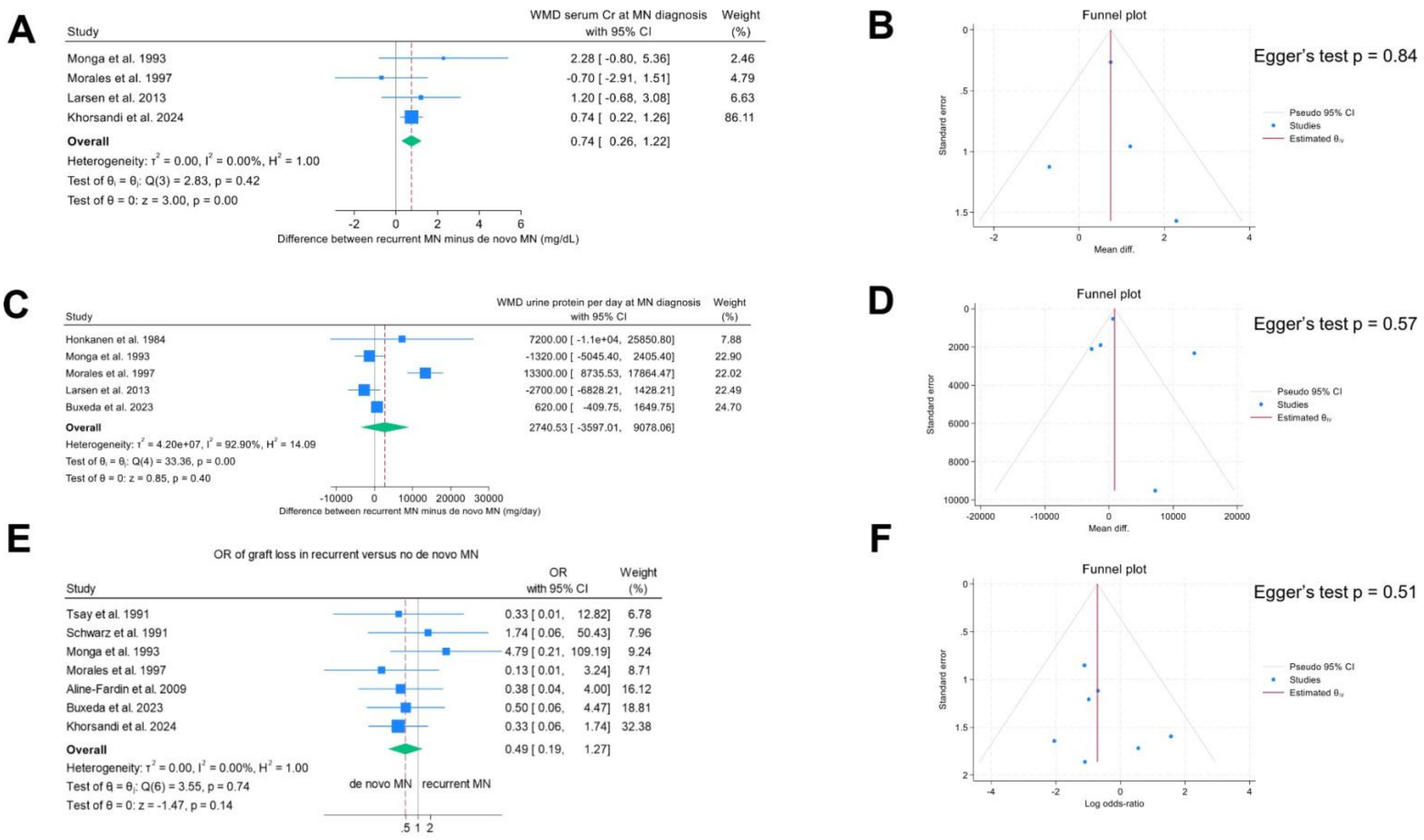

Figure S8: Forest (A) and funnel (B) plots comparing de novo membranous nephropathy with recurrent membranous nephropathy for (any) allograft rejection

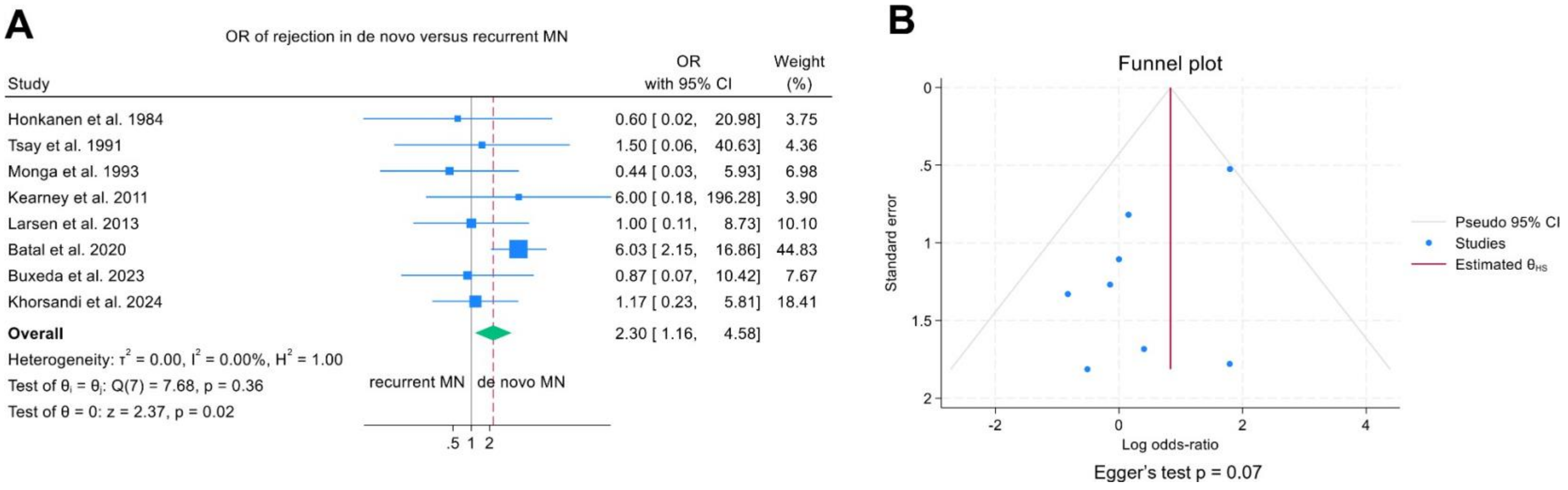

Figure S9: Funnel plots comparing recurrent MN with non-recurrent membranous nephropathy for allograft loss stratified by biopsy indication: protocol biopsies versus no protocol biopsies.

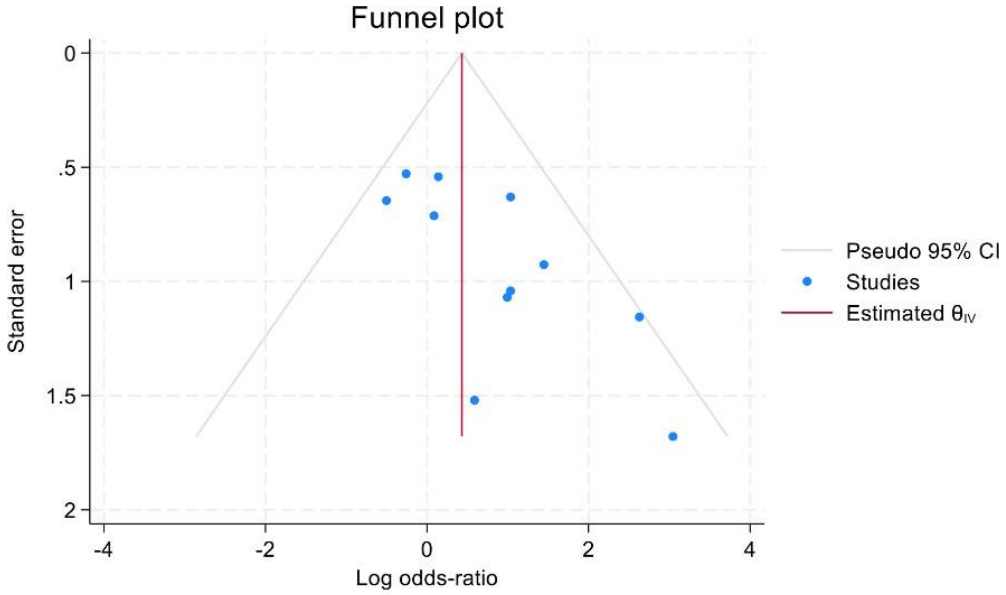

Egger’s test p = 0.009
